# Recognition of Lipopolysaccharide and Activation of NF-κB by Cytosolic Sensor NOD1 in Teleost Fish

**DOI:** 10.3389/fimmu.2018.01413

**Published:** 2018-06-26

**Authors:** Dekun Bi, Yue Wang, Yunhang Gao, Xincang Li, Qing Chu, Junxia Cui, Tianjun Xu

**Affiliations:** ^1^Key Laboratory of Exploration and Utilization of Aquatic Genetic Resources, Shanghai Ocean University, Ministry of Education, Shanghai, China; ^2^National Pathogen Collection Center for Aquatic Animals, Shanghai Ocean University, Shanghai, China; ^3^Laboratory of Fish Biogenetics & Immune Evolution, College of Marine Science, Zhejiang Ocean University, Zhoushan, China; ^4^East China Sea Fisheries Research Institute, Chinese Academy of Fishery Sciences, Shanghai, China; ^5^College of Animal Science and Veterinary Medicine, Jilin Agriculture University, Changchun, China; ^6^International Research Center for Marine Biosciences at Shanghai Ocean University, Ministry of Science and Technology, Shanghai, China

**Keywords:** NOD1, lipopolysaccharide, RIPK2, NF-κB, teleost fish

## Abstract

Lipopolysaccharide (LPS) is the major component of the outer membrane of Gram-negative bacteria. This molecule can induce strong immune response and various biological effects. In mammals, TLR4 can recognize LPS and induce inflammatory response. However, the innate receptor in fish for recognizing LPS remains ambiguous. LPS can invade the cytoplasm *via* outer membrane vesicles produced by Gram-negative bacteria and could be detected by intracellular receptor caspase-11 in mammals, so, there may also exist the intracellular receptors that can recognize LPS in fish. NOD1 is a member of NOD-like receptors family and can recognize the iE-DAP in the cytoplasm in mammals. In fish, NOD1 can also respond to infection of Gram-negative bacteria and may play an important role in the identification of bacterial components. In this study, to study whether NOD1 is a recognition receptor for LPS, we detected the expression of NOD1 and several cytokines at transcript levels to determine whether LPS can induce inflammatory response in teleost fish and NOD1 can respond to LPS. Then, we perform the binding analysis between NOD1 and ultrapure LPS by using Streptavidin pulldown assay and enzyme-linked immunosorbent assay to prove that NOD1 can be combined with LPS, and using dual luciferase reporter gene assay to verify the signal pathways activated by NOD1. Next, through cell viability analysis, we proved that LPS-induced cytotoxicity can be mediated by NOD1 in fish. The results showed that NOD1 can identify LPS and activate the NF-κB signal pathway by recruiting RIPK2 and then promoting the expression of inflammatory cytokines to induce the resistance of organism against bacterial infection.

## Introduction

Lipopolysaccharide (LPS) is a heat-stable endotoxin and is main constituent of the outer membrane of Gram-negative bacteria. This molecule has long been considered as a significant factor in septic shock (septicemia) in humans and can induce strong response from normal animal immune systems (1, 2). In mammals, LPS has been exclusively identified by TLR4 under the participation of myeloid differentiation protein 2 (MD2), LPS binding protein (LBP), and cluster of differentiation 14 (CD14) at the cell surface (3). After distinguishing LPS, TLR4 can activate certain signaling pathways, such as MyD88-dependent or MyD88-independent pathways. In MyD88-dependent pathways, TLR4 recruits MyD88 to transfer the signal and activate the NF-κB signaling pathway (4, 5). However, in MyD88-independent pathways, MD-2 (the polymer of LPS) and TLR4 forms an endosome to enter the cytoplasm and then recruits TRIF to transfer the signal induce inflammatory response (6, 7). Recent study has shown that Gram-negative bacteria can secrete outer membrane vesicles (OMVs) as an intermediary to deliver LPS into the cytosol. During Gram-negative bacteria infection, OMVs can be internalized *via* endocytosis, which then release the LPS into the cytosol from early endocytic compartments (8). Furthermore, previous studies showed that the receptors that can distinguish the LPS and activate innate immunity also exist in cytoplasm, such as caspase-4/5/11. These receptors are considered as intracellular receptors for cytosolic LPS (8–10).

Frequent outbreak of bacterial diseases in fish aquaculture results in great economic loss and is a major factor that restrains aquaculture development. Among these microbes, Gram-negative bacteria are the main pathogenic bacteria that can cause various diseases (11). As a major component of the outer membrane of Gram-negative bacteria, LPS also exhibits various biological effects. For example, LPS can induce the expression of cytokines and acute-phase proteins, and it can play an important role in pathological, neuro-immunological, and immuno-endocrinological activities in a variety of fish (12). Thus, receptors that can identify LPS should be studied extensively. TLR4 is the specific receptor in mammals for identifying LPS. However, previous studies have found that except zebrafish and several other cyprinidae, for example, rare minnow and common carp, most fish species including miiuy croaker do not exist TLR4 orthologs (13, 14). In addition, in mammals, TLR4 needs to form a tripolymer along with MD2 and the leucine-rich repeat (LRR) protein CD14 to identify LPS; however, all fish genomes lack these co-stimulatory molecules, MD2 and CD14 (15, 16). So, TLR4 in fish does not recognize the stimulation of LPS. And later studies on zebrafish TLR4 have showed that zeTLR4 cannot recognize LPS (15, 16), which directly confirms the above viewpoint. Multiple TLRs have been identified in fish and thorough analysis of the role of TLRs showed that other TLRs could also not identify LPS (15). Because of the limitations in research methods, the role of immune genes in fish was rarely investigated, thus the receptors that can recognize LPS in fish are also not clear.

NOD1 is a member of the NOD-like receptor (NLR) family, which was encoded by *CARD4* genes. This receptor is composed of an N-terminal effector binding domain (CARD), a central nucleotide oligomerization NACHT domain, and a C-terminal LRR domain (17). NOD1 is widely distributed in various tissues (18) and exists in a wide range of species. Previous studies have shown that NOD1 exists in a variety of bony fishes, such as zebrafish (19), goldfish (20), grouper (21), and miiuy croaker (22). And it also showed that miiuy croaker NOD1 is highly homologous in structure and sequence compare with many fish and mammalian species. In miiuy croaker, NOD1 can expression in all tissues, especially the high expression of NOD1 was observed in liver and skin (22). Intensive studies in mammals and fish showed that NOD1 can recognize the G-d-glutamyl-meso-diaminopimelic acid (iE-DAP) moieties that are derived from Gram-negative bacteria and then activates NF-κB signaling pathway by recruiting RIPK2 to induce inflammatory reaction (23–26). Furthermore, studies have also found that both PGN and LPS, the pathogenic components of Gram-negative bacteria, can induce significant expression of inflammatory cytokines in teleosts (27, 28). iE-DAP as the composition of PGN, it can be recognized by NOD1, so LPS may also be recognized by NOD1 in teleost fish.

Gram-negative bacteria are the main pathogenic bacteria in the majority of fish, and LPS is an important pathogenic component of these bacteria. Thus, analyzing the receptors that can detect LPS is important to study fish diseases. In this study, we use miiuy croaker (*Miichthys miiuy*) as a model fish species to study the recognition between NOD1 and LPS because of the extensive background of this species in immunology research (29–32). The results showed that both Gram-negative bacterial infection and LPS stimulation can induce inflammatory response in teleost fish, NOD1 can also respond to LPS stimulation and Gram-negative bacterial infection. The expression of inflammatory cytokines will be markedly inhibited after knockdown of NOD1 gene. Overexpression of NOD1 can activate NF-κB signal pathway, and stimulation of cells with LPS, which were overexpression of NOD1 can significantly promote the expression of NF-κB. The results of streptavidin pulldown assay and enzyme-linked immunosorbent assay (ELISA) showed that LPS can bind with NOD1 protein. Overexpression of mutant NOD1 plasmid could not activate NF-κB; simultaneous overexpression of NOD1 and RIPK2 plasmids resulted in more evident activation of NF-κB, and immunoprecipitation analysis showed that NOD1 can interact with RIPK2. These results illustrate that NOD1 can identify LPS and activate NF-κB signal pathway by recruiting RIPK2 to promote the expression of inflammatory cytokines.

## Materials and Methods

### Preparation of Tissue and Macrophage

Healthy miiuy croakers (750 ± 20 g) were obtained from Zhoushan Fisheries Research Institute (Zhejiang, China) and cultured in aerated seawater tanks at 25°C for a week. To obtain the infected tissues, the healthy fish were randomly divided into two groups: those in the experimental group were intraperitoneally injected with 1 ml *Vibrio anguillarum* (1.5 × 10^8^ CFU/ml), *Vibrio harveyi* (1.5 × 10^8^ CFU/ml), *Staphylococcus aureus* (1.5 × 10^8^ CFU/ml), and LPSs derived from *Escherichia coli* 055:B5 (1 mg/ml) and those in the control group were injected with 1 ml of physiological water. Then, the fish were dissected at different times, and the liver tissues were collected from three individual at each times.

To separate and obtain the macrophages, head kidney tissues from healthy miiuy croakers were collected and chopped, next, conduct sterile filtration by using cell filter with 100 µm pore size in L-15 medium, which was contained 2% FBS, penicillin (100 IU/ml), streptomycin (100 µg/ml), and heparin (20 U/ml). Then, the cell suspension was added into 51% Percoll (Pharmacia, USA) separating medium and centrifuged at the condition of 400 *g* at 4°C for 40 min. Next, the supernatant was removed and the cells were collected at interface, washed the cells twice with L-15 medium, and seeded in a 6-well plate at a density of about 4 × 10^7^ per well, the cells were then cultured in the incubator at 26°C with 4% CO_2_. After overnight culture, replace the medium with fresh L-15 medium, which contained 20% FBS. The cells were treated with ultrapure LPS-B5 (3 µg/ml, tlrl-pb5lps, InvivoGen), ultrapure LPS-EK (3 µg/ml, tlrl-peklps, InvivoGen), Lipoteichoic acid (LTA, 1 µg/ml, L3265, Sigma), Zymosan A (25 µg/ml, Z4250, Sigma), poly(I:C) (10 µg/ml, tlrl-picw, InvivoGen), and infected with SCRV at a multiplicity of infection (MOI) of 5, and then the cells were collected at different times. The cells without treated with any pathogenic component as the control, and each experiment will perform three biological replicates. This study was carried out in accordance with the recommendations of National Institutes of Health’s Guide for the Care and Use of Laboratory Animals. The study protocol was approved by the Research Ethics Committee of the Shanghai Ocean University (SHOU-DW-2018-047).

### Real-Time Quantitative PCR Analysis

To perform Real-time Quantitative PCR analysis, firstly, TRIzol reagent (Invitrogen) was used to extract the total RNA from macrophages and tissues, and then FastQuant RT Kit (Tiangen) was used to perform reverse transcription to avoid genomic contamination. Next, we designed the specific primers to detect the expression of NOD1, TNFα, IL-1β, IL-6, IL-8, and IFNβ in miiuy croaker, the expression of β-actin as an internal control. Using SYBR^®^ Premix Ex Taq™ (Takara) and 7300 real-time PCR system (Applied Biosystems, USA) to perform real-time quantitative PCR, the mixture of amplification containing 10 µl SYBR Premix (2×), 0.4 µl ROX Dey (50×), 0.8 µl of each primer (10 µM), 2 µl cDNA template, and 6 µl ddH_2_O. The conditions of cycle were 30 s at 95°C, followed by 40 cycles at 95°C for 5 s, and at 60°C for 34 s. Then, the dissociation curve was performed to determine the target specificity after each analysis. The triplicate experiments were performed for each sample and all the primers are listed in Table 1.

**Table 1:** PCR primer sequence information.

Primers	Sequences (5′–3′)	Application
NOD1-RT-F	TCGCACTCGTATTGGATG	Expression of NOD1
NOD1-RT-R	CACTGGTGGAAAGGTAGG	

TNFα-RT-F	GTTTGCTTGGTACTGGAATGG	Expression of TNFα
TNFα-RT-R	TGTGGGATGATGATCTGGTTG	

IL-6-RT-F	GCGGTAAAGGCATGGATAT	Expression of IL-6
IL-6-RT-R	GTTGTAGTTGGAAGGGCAG	

IL-8-RT-F	AGCAGCAGAGTCTTCGT	Expression of IL-8
IL-8-RT-R	TCTTCGCAGTGGGAGTT	

IL-1β-RT-F	CATAAGGATGGGGACAACGAG	Expression of IL-1β
IL-1β-RT-R	TAGGGGACGGACACAAGGGTA	

IFNβ-RT-F	GCTCTGCCTTCCCTGCTA	Expression of IFNβ
IFNβ-RT-R	CAGTTGACTCCGCCCTCT	

β-actin-RT-F	GTGATGAAGCCCAGAGCA	Expression of β-actin
β-actin-RT-R	CGACCAGAGGCATACAGG	

NOD1-KpnI-1F	CGGGGTACCATGTACCCATACGATGTTCCAGATTACGCTTCGTGCCTGAACATCCTC	Amplification of NOD1
NOD1-XbaI-1R	CGAGCCTCTAGACAGTCAGTGGAAGCGCAGCCT	

NOD1-GFP-N1-XhoI-1F	CCGCTCGAGATGTACCCATACGATGTTCC	Amplification of NOD1
NOD1-GFP-N1-KpnI-1R	CGGGGTACCGTGTGGAAGCGCAGCCTCTT	

RIPK2-BamHI-1F	CGCGGATCCATGGAGCCGTCCGCGGCTAT	Amplification of RIPK2
RIPK2-XhoI-1R	CCGCTCGAGACTGCCGCTACATGTTCC	

NOD1-LRR-F	TACTCAGAATTCCGTAAGAAGCTGCTCGGCCT	Amplification of NOD1-LRR
NOD1-LRR-R	TACTCACTCGAGGTGAGTGAGGGCCTCAGCCAG	

NOD1-ΔCARD-F	CCGGAATTCGAGATCAATTACAACCCAAG	Mutation of NOD1
NOD1-ΔCARD-R	CCGGAATTCGCACGAAGCGTAATCTGG	

NOD1-ΔLRR-F	CCGGAATTCAACACAGCACTCAAAGAG	Mutation of NOD1
NOD1-ΔLRR-R	CCGGAATTCGTGCTGCAGTACAAAGTTC	

NOD1-ΔLRR1-F	CCGGAATTCATGACAGTAGTGAGGTTGTG	Mutation of NOD1
NOD1-ΔLRR1-R	CCGGAATTCCACCCGCGTCTGACCAACCT	

NOD1-ΔLRR(2-4)-F	CCGGAATTCCACCCGCGTCTGACCAACCT	Mutation of NOD1
NOD1-ΔLRR(2-4)-R	CCGGAATTCTTTACACAGCTCCTCAGCG	

NOD1-ΔLRR(5-7)-F	CCGGAATTCAACACAGCACTCAAAGAG	Mutation of NOD1
NOD1-ΔLRR(5-7)-R	CCGGAATTCATGCCTCAAAGCTTCTGCG	

### Plasmid Construction

Miiuy croaker NOD1 (GenBank accession No. KP715094.1) was amplified from total cDNA by using a pair of primers with the HA tag, which were then digested with *Kpn* I and *Xba* I restriction endonucleases (Takara) and the products were connected to the vector of pcDNA3.1 (Invitrogen) between the endonucleases sites of *Kpn* I and *Xba* I. The plasmids encoding miiuy croaker TLR5s in the pFLAG-CMV-3 vector was constructed as described in Ref. (33). We designed specific primers to amplify miiuy croaker RIPK2 and NOD1 from miiuy croaker cDNA, then digest the DNA fragments, and insert into pcDNA3.1-flag and pEGFP-N1 vectors, respectively. The methods of double enzyme digestion and sequencing were used to validate the recombinant plasmids. Based on the recombinant plasmid, we designed several pairs of primers to amplify the miiuy croaker NOD1, which were deleted in different domains to construct the mutant plasmids, and named as NOD1ΔCARD, NOD1ΔLRR1, NOD1ΔLRR2-4, NOD1ΔLRR5-7, and NOD1ΔLRR. For expression of NOD1-LRR protein in bacteria, an expression system harboring the desired expression vector was constructed. We have designed a pair of primers to amplify the encoding LRRs domain of NOD1. The harvested DNA fragment was digested and inserted into pET-32a expression vector. To carry on the promoter activity analysis, NF-κB luciferase reporter plasmid was purchased from Promega and ISRE luciferase reporter plasmid was purchased from Stratagene. Endotoxin-Free Plasmid DNA Miniprep Kit (Tiangen) was used to extract the plasmids. All of the primers are listed in Table 1.

### Cell Culture, Transfection, and Luciferase Reporter Assays

Miiuy croaker kidney cell lines (MKC) were cultured in L-15 medium supplemented with 15% FBS (Gibco), 100 U/ml penicillin, 100 µg/ml streptomycin at 26°C. Epithelioma papulosum cyprini (EPC) cell lines were cultured in 199 medium that contain 10% FBS, 100 U/ml penicillin, and 100 µg/ml streptomycin under the humidified condition, at 26°C, and 5% CO_2_. HEK293 cell lines were cultured in DMEM high glucose medium that contain 100 U/ml penicillin, 100 µg/ml streptomycin, 10% FBS, and 2 mM l-glutamine under humidified condition, at 37°C, with 5% CO_2_.

Before transient transfection, cells were seeded into 24- or 12-well plates and incubated overnight. When the cell density reached about 80% of the cell culture plate, the plasmids were transfected into cells. NOD1 expression plasmid and NF-κB or ISRE luciferase reporter plasmid were co-transfected into cells to verify the role of NOD1 through luciferase reporter gene assay. After co-transfection of NOD1 expression plasmid and NF-κB reporter plasmid, the cells were stimulated with ultrapure LPS (2 µg/ml) to validate whether LPS can be detected by NOD1. The NOD1 plasmid and 100 nM NOD1-siRNA were co-transfected into cells to perform NOD1 knockdown experiment, and the NOD1 plasmid and RIPK2 plasmid along with NF-κB reporter plasmid were co-transfected to verify the interaction between NOD1 and RIPK2. The mutant plasmids of NOD1 and NF-κB reporter plasmid were co-transfected to check the role of different domain. Renilla luciferase reporter (pRL-TK, Promega) plasmid was used as the internal control and lipofectamine 2000™ reagent (Invitrogen) was used as the transfection reagent. The concentration of plasmid solution was tested by Nanodrop 2000 spectrophotometer (Thermo scientific), and all the experiments were repeated three times.

### RNA Interference

Miiuy croaker NOD1-specific siRNAs, RIPK2-specific siRNA, and miiuy croaker NOD2-specific siRNA were designed by RNAi Target Sequence Selector website (Clontech). The sequence of NOD1-siRNA1 was 5′-GAGAAAGGUGAUCAGGAAGTT-3′ (sense) and 5′-CUUCCUGAUCACCUUUCUCTT-3′ (antisense); the sequence of NOD1-siRNA2 was 5′-GGUUAACACAGAUCCCAUCTT-3′ (sense) and 5′-GAUGGGAUCUGUGUUAACCTT-3′ (antisense); the sequence of NOD1-siRNA3 was 5′-ACGAAAGUCUGGGCUUCUUTT-3′ (sense), and 5′-ACGAAAGUCUGGGCUUCUUTT-3′ (antisense). The sequence of RIPK2-siRNA was 5′-CCAUCAAGUGCCUGAAACUTT-3′ (sense) and 5′-AGUUUCAGGCACUUGAUGGTT-3′ (antisense). The sequence of NOD2-siRNA was 5′-GCUCGACCUGGUUUAUACATT-3′ (sense) and 5′-UGUAUAAACCAGGUCGAGCTT-3′ (antisense). The negative control-siRNA sequence was 5′-UUCUCCGAACGUGUCACGUTT-3′ (sense) and 5′-ACGUGACACGUUCGGAGAATT-3′ (antisense). Miiuy croaker macrophages were inoculated to 24-well plates and cultivated overnight, then transfected with 100 nM-specific siRNA into cells by using Lipofectamine 2000™ for 24 h, the cells that transfected with negative control-siRNA were used as the mock control, and then the cells were stimulated with ultrapure LPS.

### Western Blotting

To detect the expression of target gene, HEK293 cells were transfected with NOD1 expression plasmid or other expression plasmids, and after transfection of 48 h, the cells were collected using 1 × SDS loading buffer and gel electrophoresis was performed, then the semi-dry process (Bio-Rad Trans Blot Turbo System) was used to transfer the protein from gel to PVDF membrane. After blocked by 5% evaporated milk, the membrane was incubated overnight in anti-HA monoclonal antibody (Sigma) and incubated 90 min in secondary antibody that conjugated with horseradish peroxidase (Beyotime). Then, immunoreactive proteins were detected using the BeyoECL Plus (Beyotime). The digital imaging was performed by using the cold CCD camera.

### Purification of Recombinant Proteins

For NOD1-LRR protein expression in bacteria, the recombinant plasmid of NOD1-LRR was transformed into competent *E. coli* Rosetta (DE3) cells for overexpression. After induction expression with a final concentration of 0.4 mM isopropyl-β-d-thio-galactoside at 28°C for 12 h, the bacteria pellets were collected by centrifugation and resuspended in PBS containing 1% Triton X-100 for probe sonication lysis. The recombinant NOD1-LRR protein was purified using Ni-NTA His Bind Resin (QIAGEN) according to previously performed methods (34). Besides, thioredoxin (TRX) with 6 × His-tag encoded by parent vector pET-32a in *E. coli* was also expressed and used as the control.

### Streptavidin Pulldown Assay

To certify that LPS can bind to NOD1 in cells, the cells were transfected of indicated HA tagged NOD1 expression plasmids or flag-tagged TLR5s expression plasmids. After 36 h of transfection, cells were stimulated with 2 µg biotinylated ultrapure LPS that was derived from *E. coli*, O111:B4 strain (LPS-EB Biotin, tlrl-3blps, InvivoGen), and after 12 h of stimulation, cells were collected and lysed in a buffer that contain 20 mM Tris (pH7.5), 150 mM NaCl, 1% Triton X-100, and multiple protease inhibitors for 15 min. Protein extracts were incubated with 50 µl Streptavidin MagneSphere^®^ Paramagnetic Particles (SA-PMPs, Promega) for 60 min at 4°C. Unconjugated ligands were eliminated by washing the SA-PMPs-ligands complexes three times. And then, the precipitates were treated with 50 µl 1× SDS loading buffer and boiled at 95°C for 5 min followed by immunoblotting analyses.

### Binding Activity With LPS and iE-DAP

Enzyme-linked immunosorbent assay was conducted to evaluate the binding ability of NOD1-LRR to ultrapure LPS-B5, ultrapure LPS-EK, Synthetic Lipid A, iE-DAP (γ-d-Glu-mDAP, tlrl-dap, Invivogen), and β-1,3-Glucan (89862, Sigma). Each well of a microtiter plate was coated with 100 µl of 20 µg/ml ultrapure LPS, Lipid A, iE-DAP, or β-1,3-Glucan, and then incubated overnight at 37°C following a previously described protocol (35). Wells incubated with 100 µl of 50 mM Tris–HCl were used as negative control. Each well was blocked with BSA (2 mg/ml, 100 µl) for 2 h at 37°C, and then washed four times with TBST (0.05% Tween 20 in TBS). Subsequently, a series of diluted NOD1-LRR or TRX protein (0–0.6 µM in TBS containing 0.1 mg/ml BSA) were added. After incubation with recombinant protein for 3 h at room temperature, plates were rinsed four times with TBST and incubated with peroxidase-conjugated mouse monoclonal anti-His Tag antibody (1:5,000 dilution in TBS with 0.1 mg/ml BSA) at 37°C for 2 h. After rewashing four times with TBS, the plate was developed with 0.01% 3,3′,5,5′-tetramethylbenzidine (Sigma). The reaction was stopped with 2 M H_2_SO_4_, and absorbance was read at 450 nm wavelength. All assays were conducted in quintuplicate.

### Cell Viability Analysis

Adenosine triphosphate (ATP) is an important energy source for all organisms, and it can be used as an indicator of cell activity. To verify whether NOD1 can mediate cytotoxicity induced by LPS, EPC or HEK293 cells were transfected with miiuy croaker NOD1 expression plasmids or specific siRNAs and stimulated with 1 µg LPS-B5 or LPS-EK, and after stimulation of 12 h, the cells were collected to detect the concentration of ATP in the cells, then the concentration of ATP was used to represent the activity of cells. ATP bioluminescence assay kit (Promega) was used to collect the cells and GloMax 20/20 Luminometer (Promega) was used to detect the fluorescence intensity.

### Immunoprecipitation

To validate the interaction between NOD1 and RIPK2, HA-tagged NOD1 expression plasmids and flag-tagged RIPK2 expression plasmids were co-transfected into HEK293 cells. After 48 h of transfection, the cells were lysed and the protein extracts were immunoprecipitated with anti-flag antibody and Protein A + G Agarose beads overnight at 4°C on a rocker. Then, the complexes were washed three times, and 50 µl 1× SDS loading buffer was added to boil at 95°C for 5 min followed by immunoblotting analyses.

### Immunostaining and Confocal Imaging

Hela cell lines were purchased from the ATCC (Manassas, VA, USA) and cultured in DMEM high glucose medium. Hela cells were plated onto coverslips and incubated overnight, then NOD1-GFP expression plasmids and flag tagged RIPK2 expression plasmids were co-transfected into the cells. After 48 h of transfection, the cells were fixed with immunostaining fixative (Beyotime) for 30 min, washed three times with PBS, and blocked with immunostaining blocking buffer (Beyotime) for 60 min. Then, the cells were incubated in anti-flag antibody (Sigma) overnight at 4°C, followed by incubation with Cy3-conjugated anti-mouse IgG secondary antibody (Sigma) and added with the anti-fluorescence quenching reagent (Beyotime). The images were obtained with Leica TCS SP5 confocal system (Leica) equipped with 63× objective.

### Statistical Analysis

The data on relative gene expression were obtained by using the 2^−ΔΔCt^ method, and comparisons between different groups were made by one-way ANOVA Kruskal–Wallis test with Dunn’s multiple comparison test (36). All the data were represented in the form of mean ± SE, and the significant differences between different experimental groups were testified by using two-tailed Student’s *t*-test, and it was significant when the *P* < 0.05 or *P* < 0.01 versus the control groups (*n* = 3).

## Results

### LPS Can Induce the Inflammatory Response

**Figure 1:**
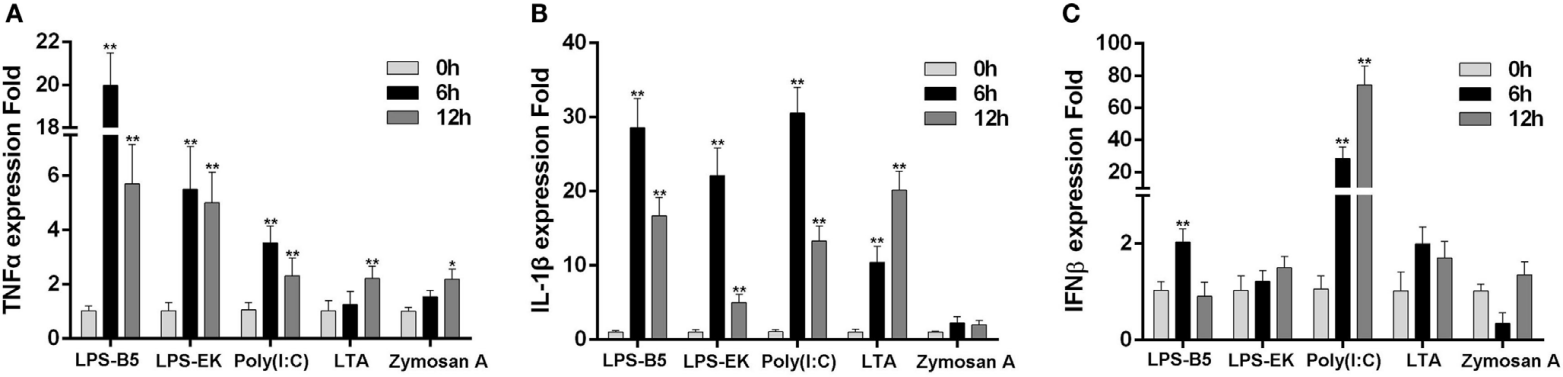
Lipopolysaccharide (LPS) can induce the inflammatory response. The expression profiles of TNFα **(A)**, IL-1β **(B)**, and IFNβ **(C)** in miiuy croaker macrophages that treated with LPS-B5, LPS-EK, poly(I:C), LTA, and Zymosan A, respectively, which were detected by using qRT-PCR. The expression of β-actin was used as the internal control, and the data were represented in the form of mean ± SE. The statistically significant differences between control and experience groups were indicated with asterisks (*P < 0.05 and **P < 0.01).

To prove whether LPS can induce the innate immune response in teleost, the expression of TNFα, IL-1β, and IFNβ in macrophages that challenged with ultrapure LPS, poly(I:C), LTA, and Zymoscan A was detected by qRT-PCR. These results showed that both LPS-B5 and LPS-EK can apparently promote the expression of TNFα and IL-1β (Figures 1A,B), but showed little effect on the expression of IFNβ (Figure 1C). Correspondingly, poly(I:C) more evidently induced the expression of IFNβ compared with TNFα and IL-1β. Compared with LPS and poly(I:C), the increase in expression of these cytokines was relatively few, which was activated by LTA and Zymosan A. These results indicated that LPS could be recognized and induce the inflammatory response in miiuy croaker.

### NOD1 Is Sensitive to the Stimulation of LPS

**Figure 2:**
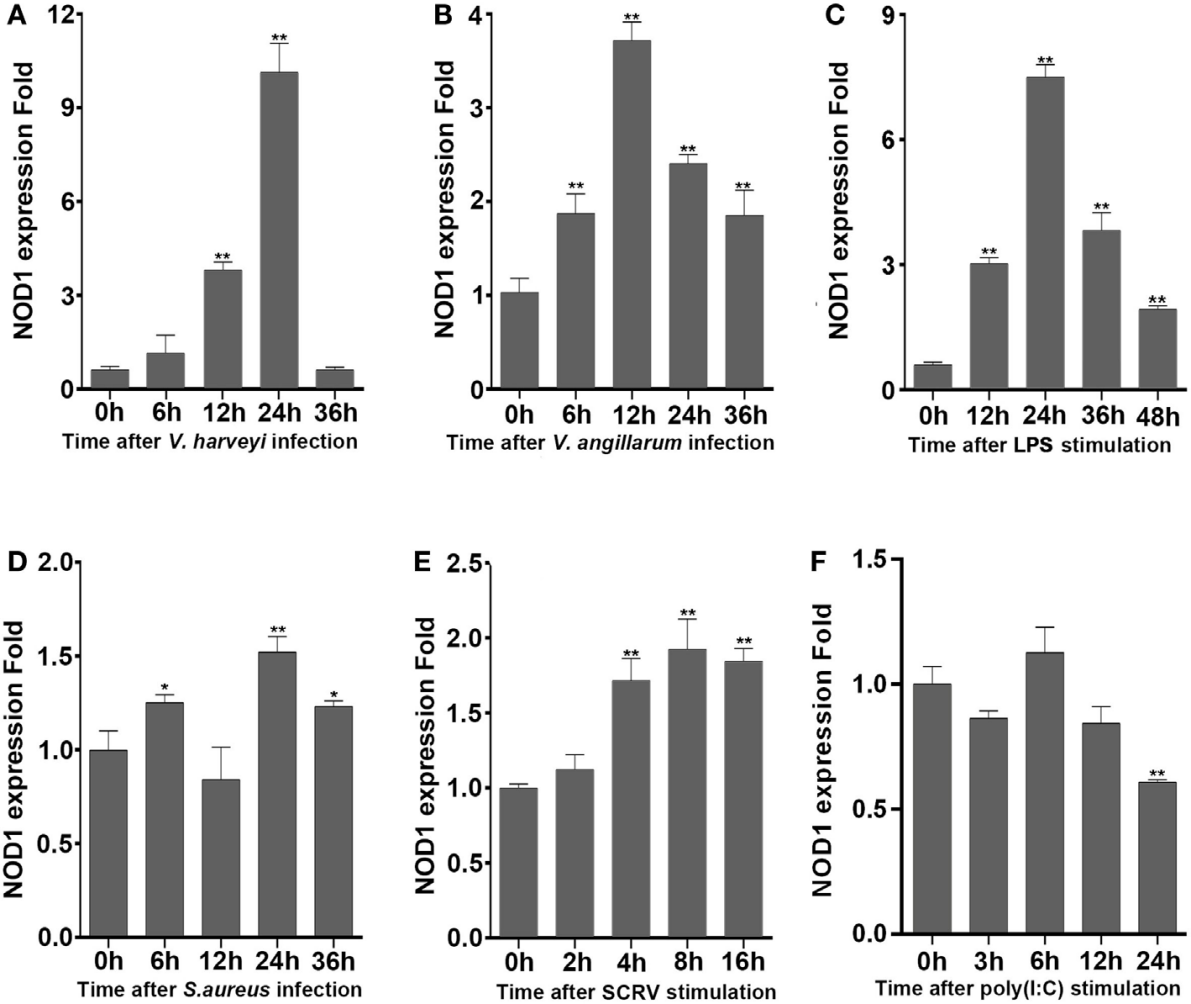
NOD1 can respond to the infection of Gram-negative bacteria. The time-course expression profiles of NOD1 were detected using qRT-PCR in miiuy croaker liver infected with *V. harvey*i **(A)**, *Vibrio anguillarum* **(B)**, lipopolysaccharide **(C)**, and *S. aureus* **(D)** and in miiuy croaker macrophages that were treated with SCRV **(E)** and poly(I:C) **(F)**. The expression of β-actin was used as the internal control, and these data were expressed in the form of mean ± SE. The statistically significant differences between control and experience group were indicated with asterisks (*P < 0.05 and **P < 0.01).

To determine whether NOD1 plays a role in the signaling pathway induced by different pathogens. The expression of NOD1 was detected in liver tissues infected with Gram-negative bacteria (*V. anguillarum* and *V. harveyi*), LPS-B5, Gram-positive bacteria (*S. aureus*), and in the macrophages challenged by SCRV, with 5 MOI which can frequently infect fish and challenged by poly(I:C), which was a synthetic analog of dsRNA. The expression of NOD1 increased remarkably in the liver after infection with *V. anguillarum, V. harveyi*, and LPS-B5 (Figures 2A–C). However, no marked increase was observed in the liver tissues infected by *S. aureus* and in the macrophages treated with SCRV or poly(I:C) (Figures 2D–F). These results demonstrate that NOD1 may be involved in Gram-negative bacteria and LPS-induced signaling pathways, but does not considerably function in the immune response induced by Gram-positive bacteria and viruses. Because NOD1 can recognize iE-DAP (23, 26) in both fish and mammal; furthermore, similar to iE-DAP, LPS is also a pathogenic component of Gram-negative bacteria. So, we can guess that NOD1 may be a recognition receptor that can identify the pathogenic components of the Gram-negative bacteria in cytoplasm.

### LPS May Be Recognized by NOD1

**Figure 3:**
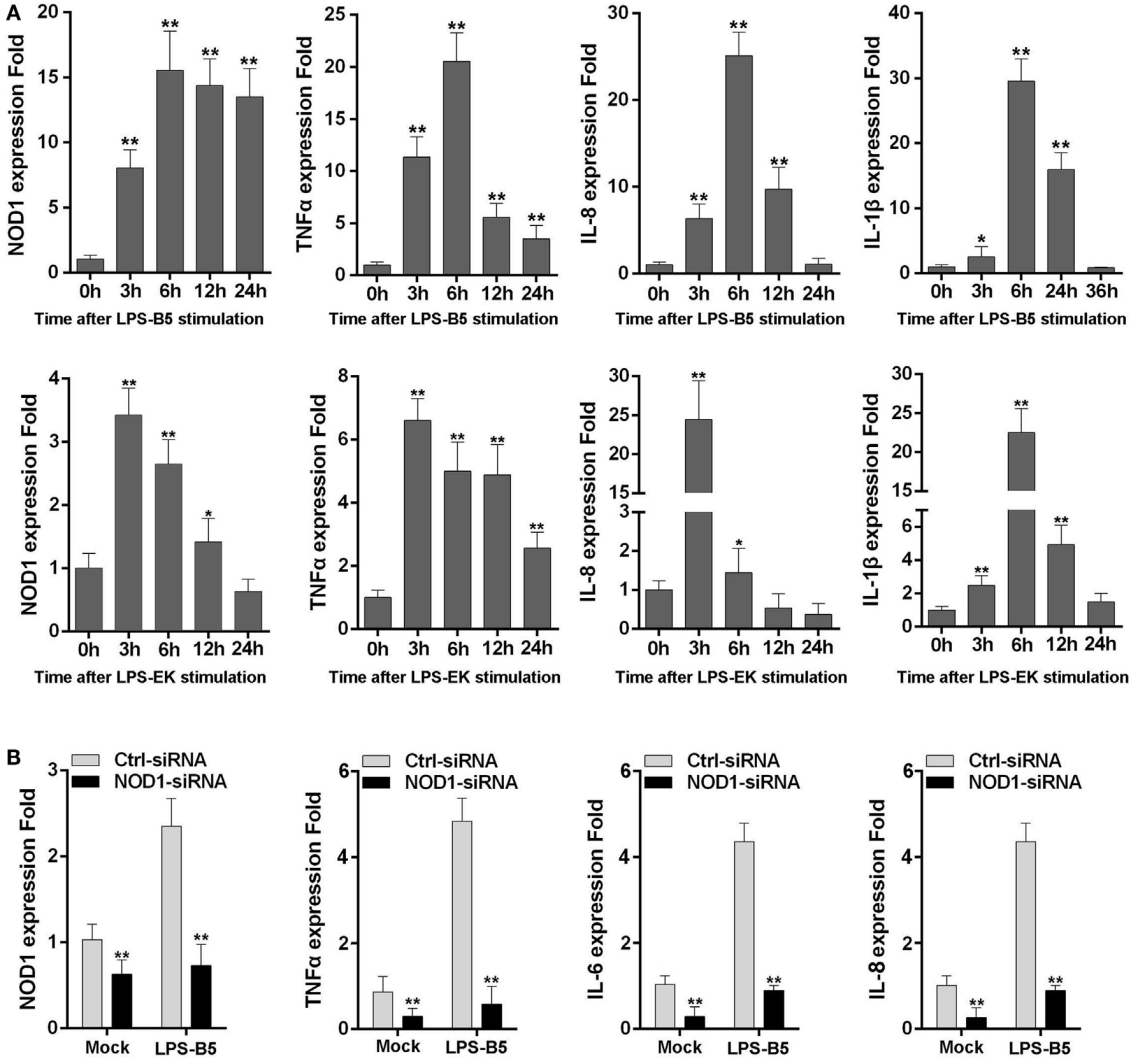
NOD1 can respond to lipopolysaccharide (LPS). **(A)** The time-course expression profiles of NOD1, TNFα, IL-8, and IL-1β in LPS-treated miiuy croaker macrophages. **(B)** Transfect the NOD1-siRNA3 into miiuy croaker macrophages, and then stimulate the macrophages by using LPS-B5, and after treating for 6 h, detect the expression of NOD1, TNFα, IL-6, and IL-8 at transcript levels in LPS-treated or untreated macrophages. The expression of β-actin was used as the internal control, and the cells transfected with negative control siRNA were used as the mock control; the data were indicated as mean ± SE. The statistically significant differences between control and experience groups are indicated with asterisks (*P < 0.05 and **P < 0.01).

The expression of NOD1, TNFα, IL-8, and IL-1β was detected in LPS-treated macrophages to further verify whether LPS can be identified by NOD1 and induce inflammatory response. Stimulation of macrophages with LPS-B5 and LPS-EK resulted in the marked increase in the expression of NOD1 and several inflammatory cytokines (Figure 3A). This result explains that LPS can probably promote the expression of NOD1 and induce inflammatory response in fish. Then, we transfected NOD1-siRNA into the macrophages and detected the expression of NOD1, TNFα, IL-6, and IL-8 before and after LPS stimulation to further verify the role of NOD1 in the identification of LPS. The result showed that NOD1-siRNA can efficiently inhibit the expression of NOD1. Moreover, after knockdown of the expression of NOD1 gene, the expression of TNFα, IL-6, and IL-8 was also decreased obviously whether or not stimulated with LPS-B5 (Figure 3B). These results can also prove that NOD1 plays an important role in the inflammatory response induced by LPS. After inhibition of the expression of NOD1 resulted in decrease in the production of inflammatory cytokines.

### NOD1 Is Sensitive to LPS and Activate the NF-κB Signal Pathway

**Figure 4:**
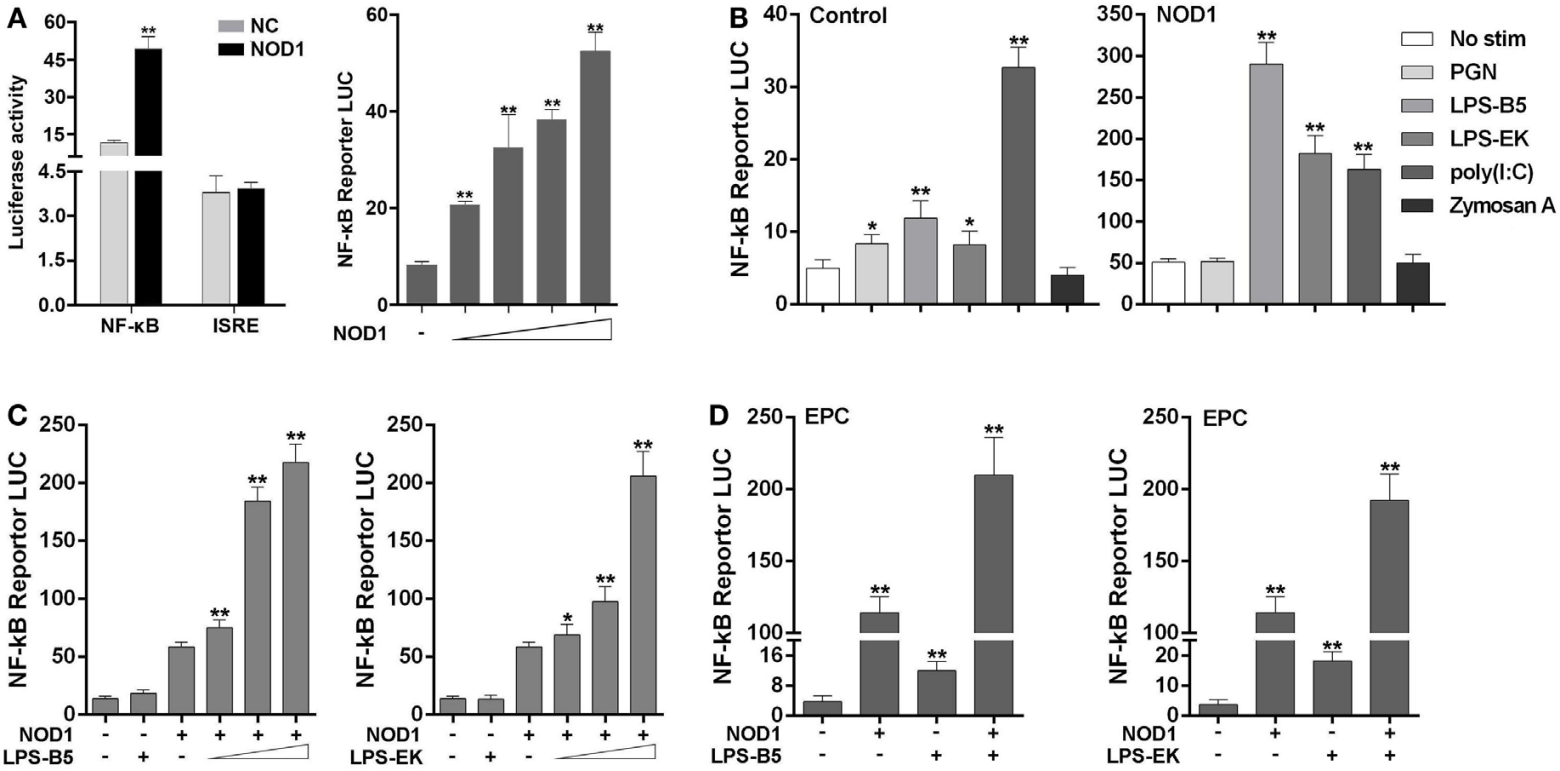
NOD1 is sensitive to lipopolysaccharide (LPS) and activate NF-κB signal pathway. **(A)** Co-transfection of miiuy croaker NOD1 expression plasmid along with NF-κB or ISRE reporter plasmid into HEK293 cells, respectively, and at 48 h after transfection, the cells were collected to detect the luciferase activity, and then transfected with different amount of NOD1 plasmids into HEK293 cells to perform a concentration gradient experiment. **(B)** HEK293 cells were transfected with NOD1 expression plasmid or empty plasmid along with NF-κB reporter plasmid, and then, cells were stimulated with PGN, LPS-B5, LPS-EK, poly(I:C), and Zymoscan A, after 12 h of stimulation, cells were collected to detect the luciferase activity. **(C)** The concentration gradient experiment of LPS-B5 and LPS-EK. **(D)** EPC cells were transfected with NOD1 expression plasmid or empty plasmid along with NF-κB reporter plasmid, then cells were stimulated with LPS, after 12 h of stimulation, the cells were collected to detect the luciferase activity. All the experiments were co-transfected with Renilla luciferase reporter (pRL-TK, Promega) plasmid as the internal control, the data were represented as mean ± SE. The statistically significant differences between control and experience groups were shown with asterisks (*P < 0.05 and **P < 0.01).

The signal pathway that can be activated by NOD1 was determined by co-transfecting NOD1 expression plasmids and NF-κB or ISRE reporter plasmids into HEK293 cells, pRL-TK plasmids as the internal control, and then dual luciferase reporter gene assay was performed (Figure 4A). The result showed that compared with the negative control, NOD1 can significantly activate the expression of NF-κB. Then, a concentration gradient experiment was performed to further verify the result. The findings indicated that NOD1 may induce the immune response by activating the NF-κB signal pathway. To confirm whether NOD1 can recognize LPS, HEK293 cells were co-transfected with NOD1 expression plasmid and NF-κB reporter plasmid. Then, the cells were stimulated with different ligands, and the luciferase activity was detected after 12 h of stimulation (Figure 4B). The result showed that after stimulating the cells with various pathogens, only LPS-B5 and LPS-EK could not markedly promote the NF-κB luciferase activity without overexpression of NOD1 but can significantly activate the NF-κB luciferase activity after overexpression of NOD1. To further verify this result, a concentration gradient experiment of ultrapure LPS-B5 and LPS-EK in HEK293 cells (Figure 4C) and the dual luciferase reporter assay in EPC cells (Figure 4D) were performed. Results showed that after stimulated with LPS-B5 and LPS-EK, over-expression of NOD1 can substantially promote the NF-κB luciferase activity both in HEK293 cells and in EPC cells. To identify the location of NOD1 distinguish LPS, HEK293 cells were transfected with NOD1 expression plasmid and NF-κB reporter plasmids, pRL-TK plasmids as the internal control. Then perform intracellular and extracellular stimulation by using LPS-B5 and LPS-EK (Figure S7 in Supplementary Material). Collect the cells to detect Luciferase activity. Result showed that NOD1 can identify the stimulation of LPS in the cytoplasm. Therefore, from the above results, we believe that NOD1 may be the intracellular recognition receptor for LPS.

### Specific Binding Activity to LPS and iE-DAP

**Figure 5:**
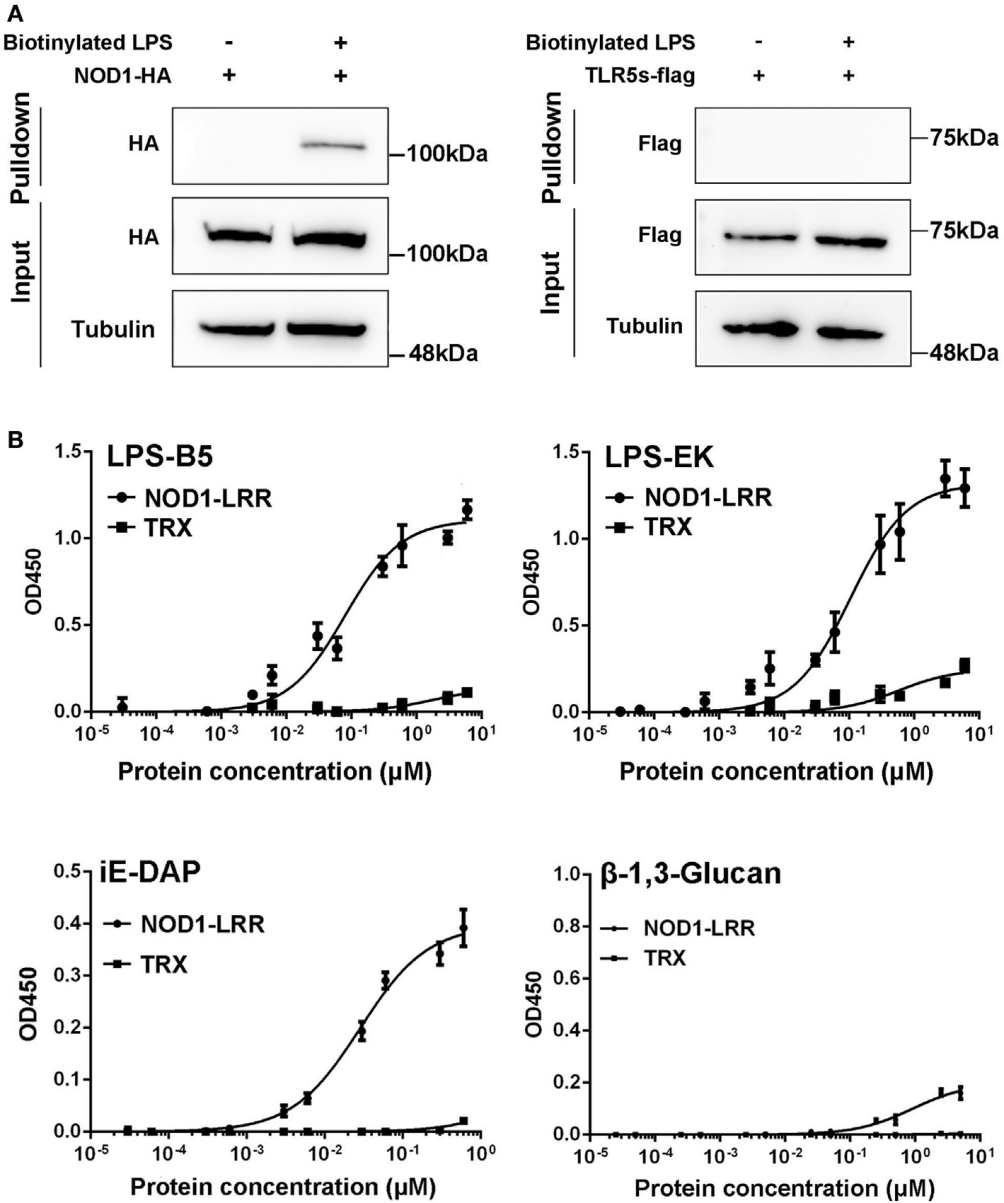
Binding activity analysis of NOD1-LRR protein to lipopolysaccharide (LPS) and iE-DAP. **(A)** Streptavidin pulldown assays of LPS binding to endogenous NOD1 and TLR5s. The cropped HA and Flag blot are shown for pulldown and input, a cropped Tubulin blot is shown for input. For um-cropped blot, see Figures S3A,B in Supplementary Material. **(B)** Binding activity analysis of miiuy croaker NOD1-LRR and TRX to ultrapure LPS-B5, LPS-EK, iE-DAP, and β-1,3-Glucan by using enzyme-linked immunosorbent assay. For un-cropped NOD1-LRR and TRX blot, see Figure S3C in Supplementary Material.

To further reveal the likely physiological function of NOD1 in fish, streptavidin pulldown assay was performed. As shown in Figure 5A and Figure S2A in Supplementary Material, miiuy croaker NOD1 proteins can be pulled down by SA-PMPs (Streptavidin MagneSphere^®^ Paramagnetic Particles, Z548, Promega) through biotinylated ultrapure LPS. On the contrary, miiuy croaker TLR5s protein, which was a recognition receptor of the TLR family (33), cannot be pulled down by SA-PMPs. So, it can be proved that miiuy croaker NOD1 can combine with LPS in cells.

Then, to further validate the binding effect between NOD1 and LPS, ELISA was performed. Although we used ultrapure LPS-B5and LPS-EK in experiments, in order to further eliminate the interference from other components, we also did the binding experiments between NOD1 and Lipid A. Lipid A is the innermost of the three regions of the LPS, and many immune-activating abilities of LPS can be actually attributed to lipid A. Thus, the binding between NOD1 and Lipid A was detected to further confirm the binding between LPS and NOD1 (Figure S2B in Supplementary Material). In addition, iE-DAP is recognized as a ligand for NOD1 in mammal and fish (23, 26), considering that the LRR domain of NOD1 is the binding domain for iE-DAP (25). We detected the binding activity of NOD1–LRR with iE-DAP as the positive control to confirm the binding activity of NOD1-LRR with LPS. At the same time, because β-1,3-Glucan was the ligand that cannot be recognized by NOD1, the binding activity of NOD1–LRR with β-1,3-Glucan was also detected as a negative control. These results revealed that NOD1–LRR could strongly bind to ultrapure LPS, Lipid A, and iE-DAP in a concentration-dependent manner, but could not bind to β-1,3-Glucan. Moreover, NOD1–LRR possessed stronger binding ability to ultrapure LPS and Lipid A than to iE-DAP, because NOD1–LRR showed apparent binding activity to LPS and Lipid A at a low concentration (less than 0.1 nM). By contrast, significant binding activity with iE-DAP required higher NOD1–LRR concentration (more than 1 nM) (Figure 5B). Additionally, NOD1–LRR harvested higher optical density values for the binding to ultrapure LPS and Lipid A than to iE-DAP at the same NOD1–LRR concentration. These results also demonstrated that NOD1-LRR possessed more potent binding activity with LPS. By contrast, TRX exhibited very weak binding activity with ultrapure LPS, Lipid A, iE-DAP, or β-1,3-Glucan. These findings suggest that NOD1 is the receptor for iE-DAP (25), and NOD1 could be a potential receptor for LPS.

### Knockdown of NOD1 Reduces NF-κB Activity

**Figure 6:**
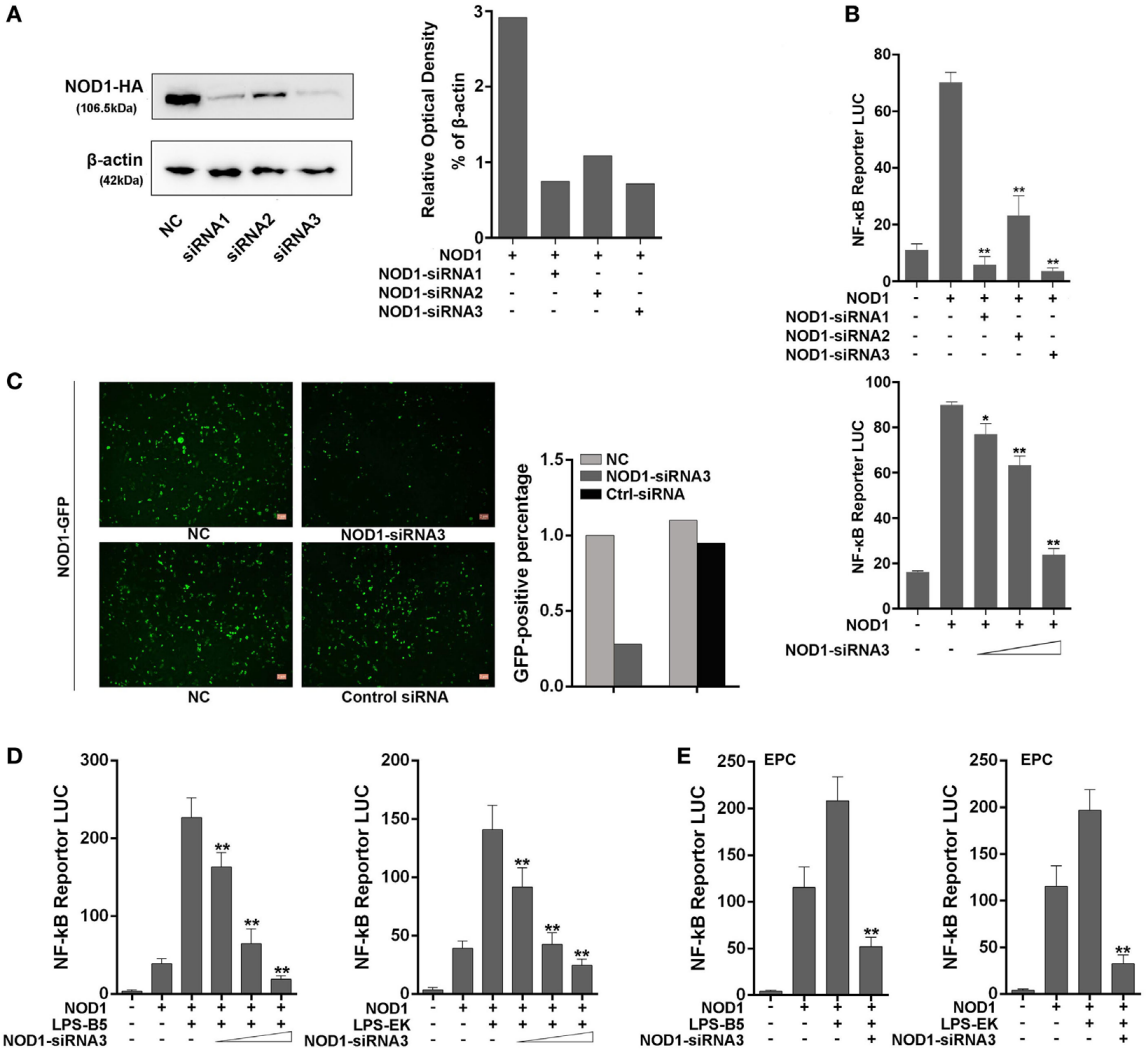
NOD1-siRNA inhibits the role of NOD1 in recognizing lipopolysaccharide (LPS). **(A)** Miiuy croaker NOD1 expression plasmid was co-transfected into HEK293 cells along with three NOD1-siRNA or control siRNA, respectively, after 48 h of transfection, the cells were collected to perform western blotting analysis and the relative optical density value assay were performed according to the western blotting analysis results. A cropped NOD1 and β-actin blot are shown. For un-cropped NOD1 and β-actin blot, see Figure S4 in Supplementary Material. **(B)** HEK293 cells were co-transfected with NOD1 expression plasmid and NF-κB reporter plasmid along with three NOD1-siRNA, then the luciferase activity was detected. Next, co-transfection of NOD1 expression plasmid and NF-κB reporter plasmid along with different quantity of NOD1-siRNA3 to perform a concentration gradient experiment. **(C)** HEK293 cells were co-transfected with miiuy croaker NOD1-GFP plasmid and NOD1-siRNA3 or control siRNA to detect the fluorescence quantity. **(D)** Co-transfection of NOD1 expression plasmid and NF-κB reporter plasmid along with NOD1-siRNA3 into HEK293 cells, then stimulate the cells with LPS-B5 and LPS-EK to perform concentration gradient experiment. **(E)** Co-transfection of NOD1 expression plasmid and NF-κB reporter plasmid along with NOD1-siRNA3 into EPC cells, then stimulate the cells with LPS-B5 and LPS-EK to perform dual-luciferase reporter gene assay. The pRL-TK plasmid was used as the internal control, and data are expressed as mean ± SE. The statistically significant differences were indicated by asterisks (*P < 0.05 and **P < 0.01).

Three siRNAs were designed and transfected into cells to knockdown NOD1 to further examine the role of NOD1 in the inflammatory response induced by LPS. Moreover, different experimental techniques were used to validate the efficiency of these siRNAs. Western blot result and the relative optical density value clearly showed the inhibitory effect of NOD1-siRNA on the expression of NOD1 (Figure 6A). Then, NOD1 expression plasmids and NF-κB reporter plasmids were co-transfected with the three NOD1-siRNAs into HEK293 cells to further test the role of NOD1-siRNA by detecting the luciferase activity. Figure 6B showed that all of the three NOD1-siRNAs can play a significant inhibitory role on the expression of NOD1. However, the role of NOD1-siRNA3 was more obvious compared with the other two siRNAs. NOD1-GFP recombinant plasmid can express the NOD1 protein with green fluorescence protein. Thus, transfection of NOD1-GFP plasmid along with NOD1-siRNA3 or control siRNA into cells was performed, then, the intensity of fluorescence was detected to validate the efficiency of NOD1-siRNA3 again. As shown in Figure 6C, result showed that NOD1-siRNA3 could effectively inhibit the expression of NOD1. Finally, a concentration gradient of LPS-B5 and LPS-EK and the dual luciferase reporter gene assay in EPC cells were performed to demonstrate the role of NOD1 in the recognition of LPS (Figures 6D,E). After inhibiting the expression of NOD1, the activity of NF-κB induced by LPS was also greatly reduced. These results indicate that NOD1 may be a receptor, which can recognize LPS in cells.

### NOD1 Can Identify LPS and Mediates Cytotoxicity

**Figure 7:**
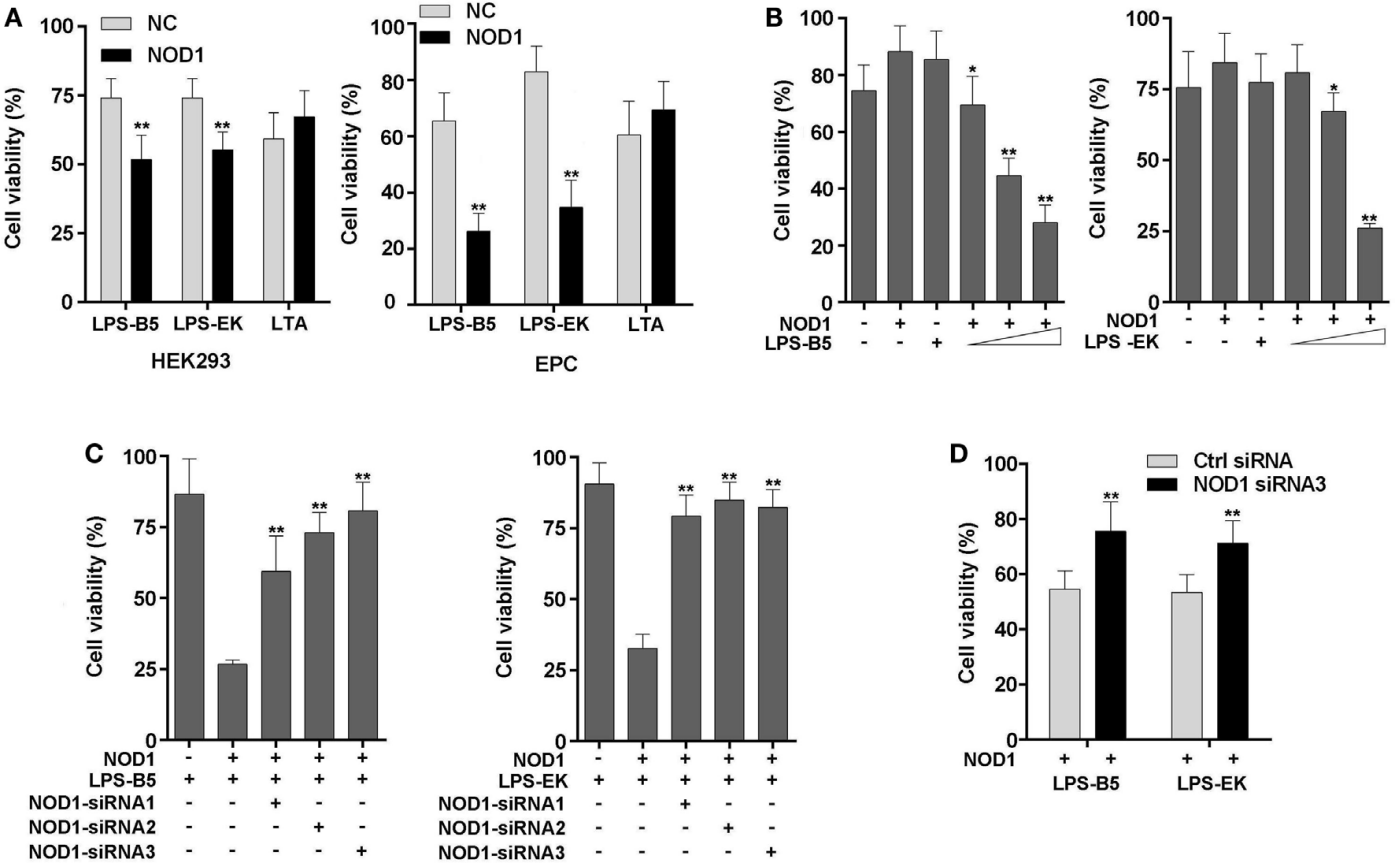
NOD1 mediates cytoplasmic lipopolysaccharide (LPS)-induced cytotoxicity in cells. **(A)** HEK293 and EPC cells were transfected with NOD1 expression plasmid, then stimulated the cells with LPS-B5, LPS-EK, or LTA, next after 12 h of stimulation, collect the cells to detect the cell viability. **(B)** NOD1 expression plasmid was transfected into HEK293 cells and stimulated the cells to perform concentration gradient test. **(C)** HEK293 cells were co-transfected with NOD1 expression plasmid and different NOD1-siRNAs, after 36 h of transfection, the cells were stimulated with LPS-B5 and LPS-EK, then collect the cells and detect the cell viability. **(D)** NOD1-siRNA3 were transfected into EPC cells that were over-expression NOD1, and then the cells were stimulated with LPS-B5 and LPS-EK, and the cell viability was detected after 12 h of stimulation. The data were expressed as mean ± SE. The statistically significant differences were indicated by asterisks (*P < 0.05 and **P < 0.01).

To further confirm that miiuy croaker NOD1 can identify LPS, cell viability was analyzed by detecting the concentration of ATP in HEK293 cells and EPC cells. Results showed that after stimulation with LPS-B5 and LPS-EK, the cell viability will decrease significantly in both HEK293 and EPC cells with over-expression NOD1, but the cell activity only have little change after being stimulated with LTA (Figure 7A). Moreover, as shown in Figure 7B, we can find that this change is concentration dependent. Then, co-transfected of NOD1 expression plasmid and NOD1-siRNA into cells to perform knockdown analysis, results showed that both in HEK293 and EPC cells, after stimulated with LPS, knockdown of NOD1, the cell activity will raise substantially (Figures 7C,D), this indicated that knockdown of NOD1, the cytotoxicity induced by LPS will be unable to affect the cell viability. So, from the above results, we can consider that NOD1 can identify LPS and mediate LPS-induced cytotoxicity in fish cells.

### NOD1 Activates NF-κB Signaling Pathway by Recruiting RIPK2

**Figure 8:**
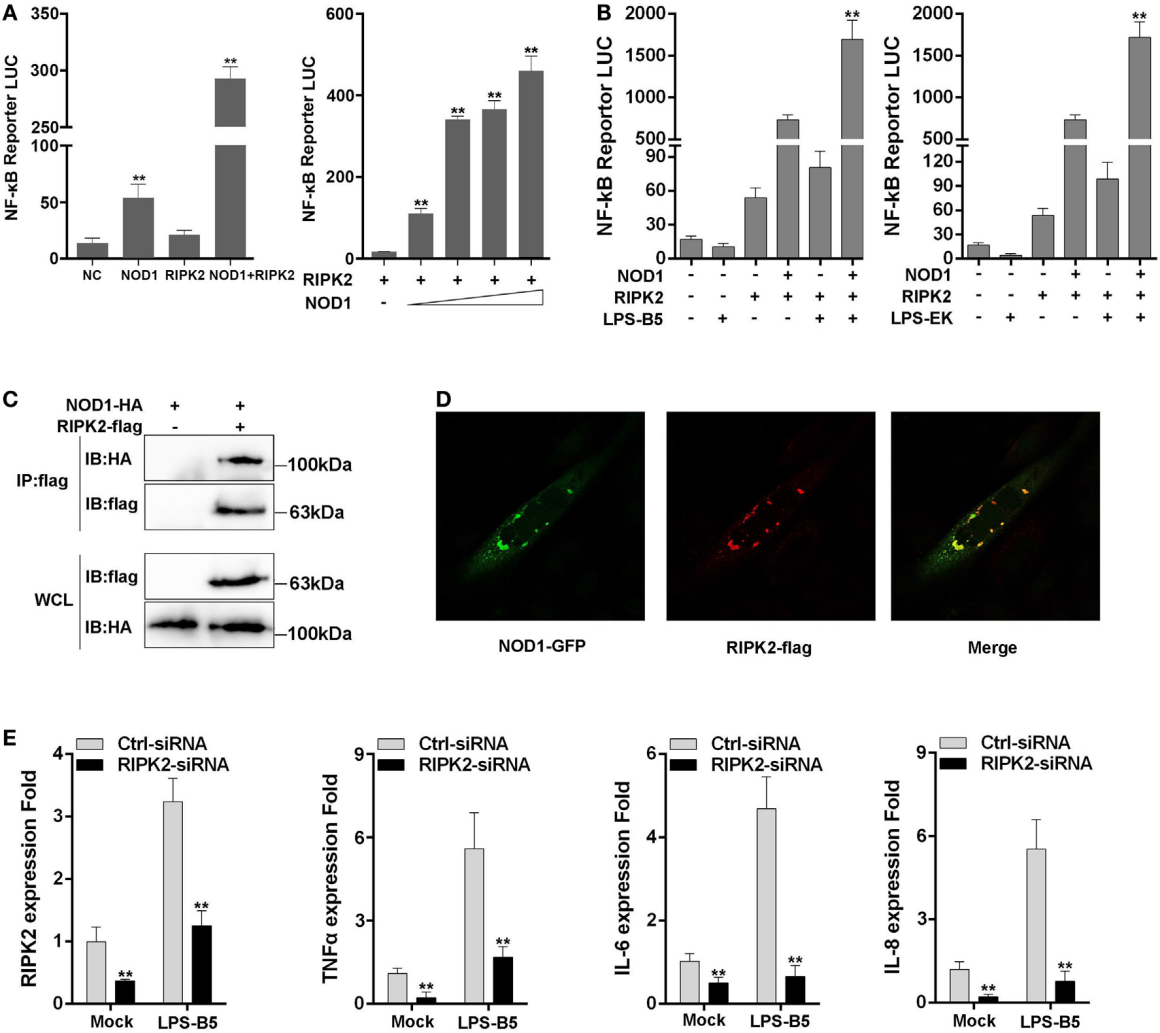
NOD1 recruits RIPK2 to activate the NF-κB signaling pathway. **(A)** Co-transfection of miiuy croaker RIPK2 and miiuy croaker NOD1 expression plasmids into HEK293 cells, and after 48 h of transfection, the luciferase activity was detected. Next, co-transfection of RIPK2 and different amount of NOD1 expression plasmids into HEK293 cells to perform a concentration gradient experiment, pRL-TK plasmids was used as the internal control. **(B)** HEK293 cells, which have been transfected with NOD1 and RIPK2 plasmids, were stimulated with lipopolysaccharide (LPS)-B5 and LPS-EK, pRL-TK plasmids were used as the internal control, and after 12 h of stimulation, cells were collected and luciferase activity was detected. **(C)** Immunoprecipitation analysis of NOD1 and RIPK2. A cropped HA blot is shown for IP and input, a cropped Flag blot is shown for input. For un-cropped HA and Flag blot, see Figure S5 in Supplementary Material. **(D)** Immunostaining and confocal imaging experiment of NOD1 and RIPK2. **(E)** Transfection of RIPK2-siRNA into miiuy croaker macrophages, which were treated with LPS-B5, and the expression of RIPK2, TNFα, IL-6, and IL-8 were detected. The data were represented as mean ± SE, and the statistically significant differences were expressed with asterisks (*P < 0.05 and **P < 0.01).

RIPK2 is the receptor-interacting protein of NOD1 and NOD2, and this protein has been confirmed in mammals. To determine whether NOD1 was also needed to recruit RIPK2 through protein–protein interaction to transfer the signal in fish, RIPK2 expression plasmids were constructed and co-transfected with NOD1 plasmids into cells for luciferase activity assay. The results showed that the activation of NF-κB was significantly increased when the cells were co-transfected with RIPK2 and NOD1 plasmids compared with the experiment that singlely transfected with NOD1 or RIPK2 plasmid. Then, we performed a concentration gradient experiment of NOD1 to further verified this result (Figure 8A). Next, the cells were transfected with NOD1 and RIPK2 plasmids along with NF-κB reporter plasmids and then stimulated with LPS-B5 and LPS-EK, after 12 h of stimulation, the cells were lysed and the fluorescence activity was detected. As shown in Figure 8B, LPS can induce significant increase in NF-κB activity after co-transfection of NOD1 and RIPK2 plasmids. To validate the protein–protein interaction between NOD1 and RIPK2, immunoprecipitation and confocal imaging were performed, the results can be unambiguously proved that NOD1 and RIPK2 can be combined directly to transmit the signal and activate the downstream signaling pathway (Figures 8C,D).

Then, to further verify the influence of RIPK2 for signal transduction in fish, the macrophages were transfected with RIPK2-siRNA to silence the expression of RIPK2. Then, the cells were stimulated with LPS-B5 and the expression of RIPK2, TNFα, IL-6, and IL-8 were detected by using qRT-PCR (Figure 8E). Result showed that after inhibition of the expression of RIPK2, the expression of TNFα, IL-6, and IL-8 were also suppressed. These results illustrate that similar to mammals, NOD1 was also needed to recruit RIPK2 to transfer the signal to induce inflammatory response in fish.

### NOD1 Recognition of LPS Rely on LRR Domain

**Figure 9:**
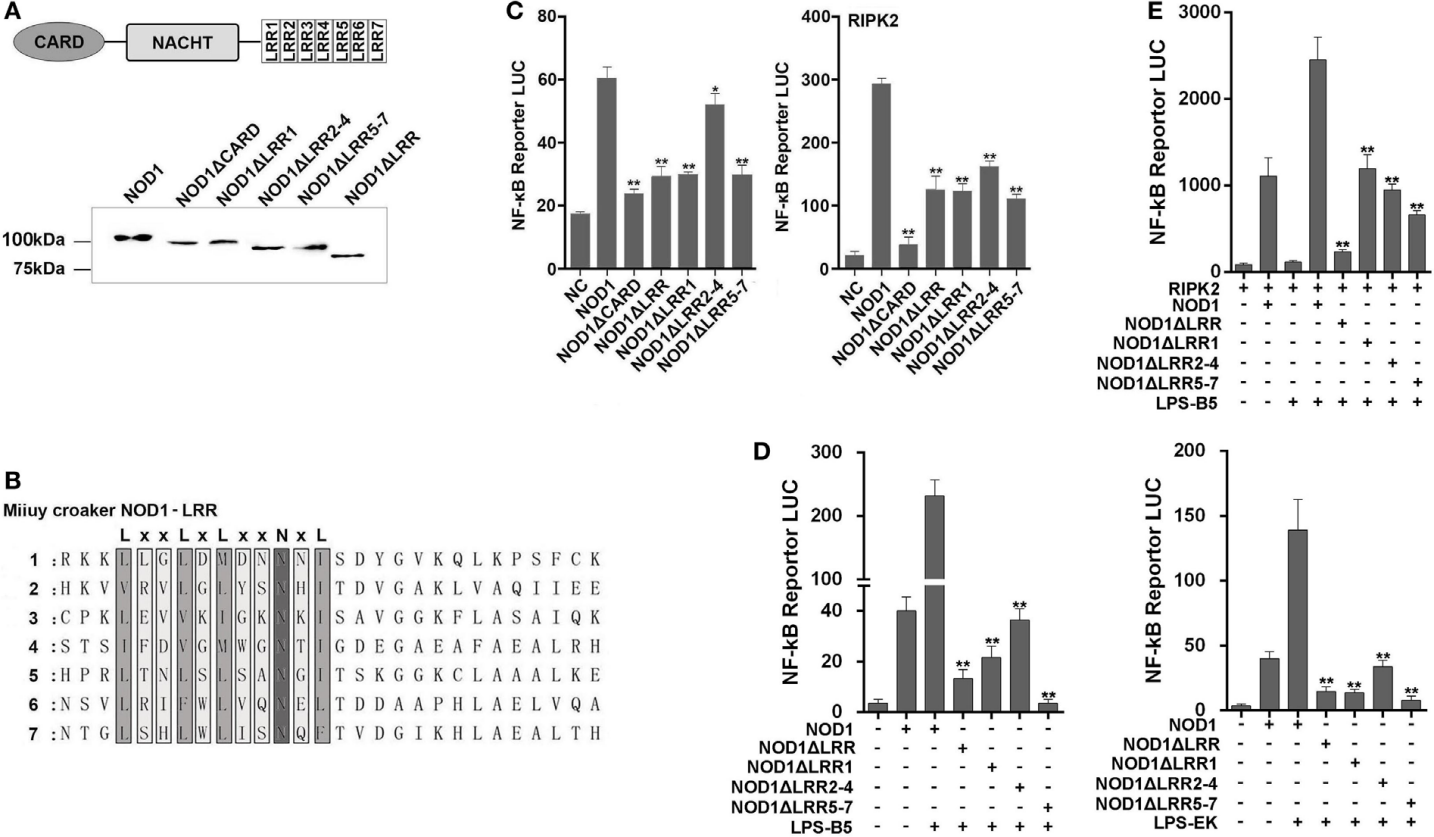
Mutant NOD1 cannot recognize lipopolysaccharide (LPS) and activate NF-κB signaling pathway. **(A)** HEK293 cells were transfected with wild type and mutant miiuy croaker NOD1 plasmids, respectively, and after 48 h of transfection, the cells were cleaved and western blotting analysis was performed. A cropped NOD1 and several mutant NOD1 blot are shown. For un-cropped blots, see Figure S6 in Supplementary Material. **(B)** The sequence analysis of miiuy croaker NOD1-LRR domain. **(C)** HEK293 cells were co-transfected with wild type or mutant NOD1 plasmids along with NF-κB reporter plasmid and miiuy croaker RIPK2 plasmid, pRL-TK plasmid was used as the internal control, after 48 h of transfection, and the luciferase activity was detected. **(D)** The cells were co-transfected of wild type or mutant NOD1 plasmids along with NF-κB reporter plasmid, pRL-TK plasmid was used as the internal control, then the cells were stimulated with LPS-B5 and LPS-EK, after 12 h of stimulation, the cells were collected to detect the luciferase activity. **(E)** HEK293 cells were transfected with NOD1 or mutant expression plasmid along with NF-κB reporter plasmid and RIPK2 expression plasmids, next, the cells were stimulated with LPS-B5, after 12 h of stimulation, cells were collected to detect the luciferase activity. All data were shown as mean ± SE, and the statistically significant differences were indicated by asterisks (*P < 0.05 and **P < 0.01).

We constructed different mutant plasmids to verify the function of the different domains of NOD1. The schematic of NOD1 mutant plasmids was displayed in Figure S1 in Supplementary Material. First, the cells were transfected with the wild-type or mutant NOD1 plasmids to examine the MW and confirm the expression (Figure 9A). Given that NOD1 was structurally homologous with the mammalian NOD1, the same domains may have the same role in the identification of ligands. In general, the LRRs domain was used to identify ligands, and a study has shown that a conserved motif of “LxxLxLxxNxL” exists in the LRR sequence of miiuy croaker NOD1 (Figure 9B) (29). Accordingly, LRRs domain may also play the role of recognizing ligands in teleost fish. Then, we determine the role of different domains of miiuy croaker NOD1 by transfecting the wild-type or mutant NOD1 plasmids into cells along with NF-κB reporter plasmids and perform dual luciferase reporter gene assay. Results showed that the expression of NF-κB that was activated by mutant NOD1 plasmids was far less than that activated by wild-type NOD1 plasmid. These results imply that all the domains will play an essential role in activating the NF-κB signal pathway (Figure 9C). Then, we transfected the mutant plasmids, which were mutated in the LRR structure, along with NF-κB reporter plasmids and RIPK2 plasmid into cells, and treated the cells with LPS-B5 and LPS-EK to further detect the function of LRR domains in the identification of LPS. Figures 9D,E shows that, whether or not existence of RIPK2, after recognizing LPS, the expression of NF-κB that activated by mutant NOD1 plasmids which lack one or several LRR structures was obviously reduced compared with activated by wild-type NOD1 plasmids. These results mean that LRR domains were the major areas of recognition, and deletion of any area can cause NOD1 to lose its recognition function.

**Figure 10:**
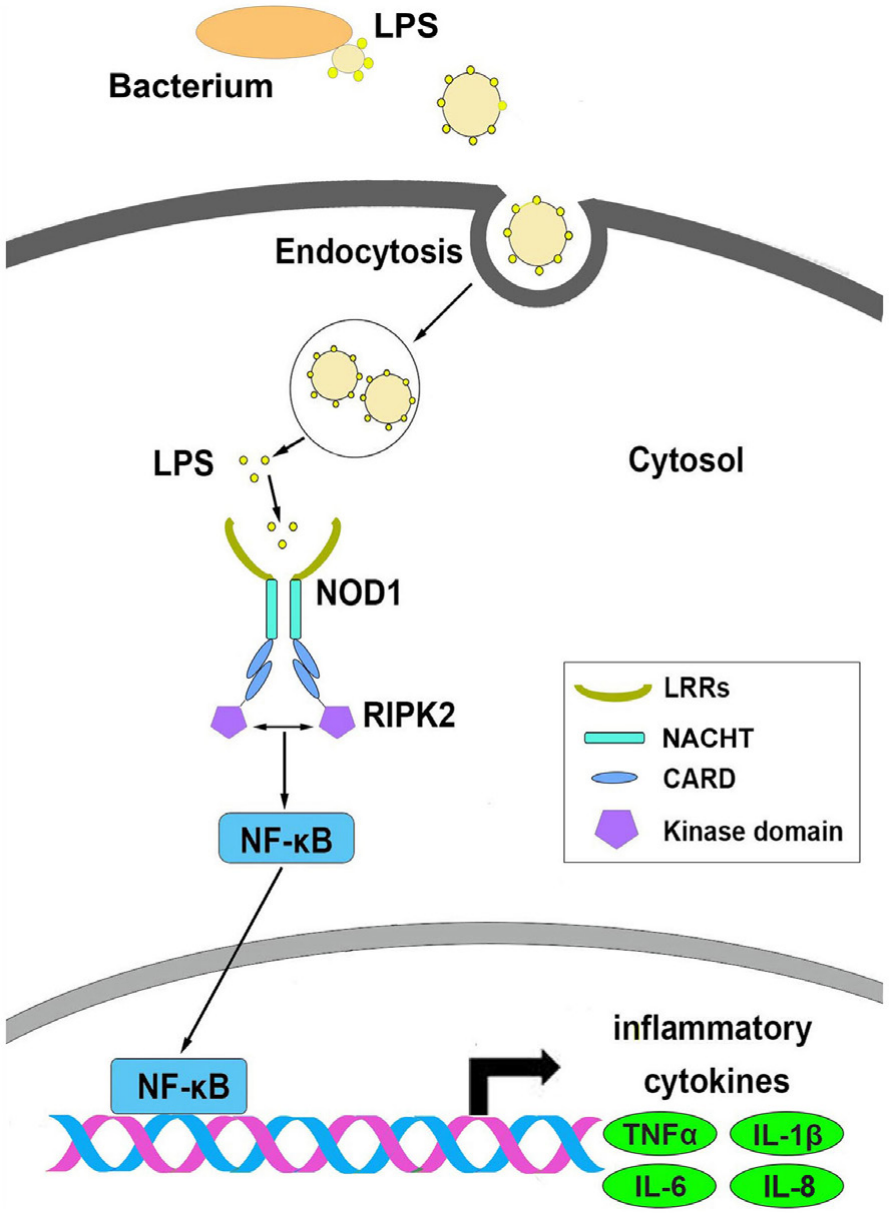
The possible signal pathways that explain the process of NOD1 recognition of lipopolysaccharide and activate NF-κB signal pathway in miiuy croaker.

Based on the above results, we conceive a possible signal pathway to explain the process of NOD1 recognition of LPS and induction of inflammatory response (Figure 10). First, LPS was secreted from Gram-negative bacteria and then invaded to the cytosol, and after secretion into the cytoplasm, LPS could be identified by NOD1 through the LRR domain, and then through CARD domain to recruit RIPK2 to transfer the signal. This process was followed by activation of the NF-κB signal pathway to induce the expression of inflammatory cytokines.

## Discussion

Fish being the lower vertebrate is a favorable animal material to study the function evolution of the immune system. Comparison of the immune genes between fish and mammals shows certain difference in both quantity and function. For example, in mammals, 13 TLRs have been characterized and showed to identify the specific pathogen-associated molecular pattern (37). However, at least 18 TLR types have been identified in various species of fish (13, 38), and TLR18–20 and TLR23–28 are only found in fish (39, 40). Furthermore, TLR6 and TLR10 were not found in fish, but TLR14 plays functions similar to TLR6 and TLR10 in identifying various pathogens and activating immune response (41). Thus, the function of the same gene in fish may not play the same role compared with the genes in mammals. In mammals, TLR4 is a central protein that distinguishes LPS. By contrast, in fish, TLR4 is only found in several kinds of fish and cannot identify LPS (15). Thus, other methods to identify LPS should be explored.

In the mammal immune system, the complex of TLR4, CD14, and MD2 has been proved to be the receptor for LPS at the cell surface (3). And several intracellular LPS receptors, such as caspase-4/5/11, are also present in the cytoplasm (9). These receptors can play an important role against invading bacteria. For example, epithelial cells were insensitive to extracellular LPS. However, LPS can also activate the NF-κB signal in the cytoplasm of these cells (42). This characteristic indicates that, in some epithelial cells which are located in particular tissues, such as the gut, because these cells are often exposed to bacteria or bacterial products; however, the expression of cell surface receptors in these cells is very low, the intracellular receptors will play an important role in resistance against bacterial infection (43, 44). In this study, we demonstrated that NOD1, as an intracellular receptor, can identify LPS and activate the NF-κB signal pathway to induce inflammatory response in teleost fish. In aquaculture, fish gill and gut are always exposed to large amounts of bacteria or bacterial products, especially Gram-negative bacteria. To date, the receptors on the cell surface of fish that can identify LPS remain unclear. Thus, NOD1 shows unparalleled importance in the resistance against bacterial infections in fish. In addition, from Figure 3B, we can find that after silencing the expression of NOD1 by using NOD1 siRNA, the stimulation of LPS can still significantly increase the expression of inflammatory cytokines, this shows that other receptors still exist in fish similar to in mammals to recognize LPS and activate the inflammatory response. Further research is required to determine whether these receptors also play an important role in fish.

NOD-like receptor is a promising intracellular recognition receptor family. Recent reports have indicated that several members of this family exist in the lower vertebrates (45) and in invertebrates (46). Many studies on this gene family have been conducted in mammals. However, few related studies have been performed in other vertebrates, such as birds, amphibians, and fish (22), resulting in little to no information about NLRs in these species (47). NOD1 is one of the representative members of the NLR family. This molecule has been studied extensively in mammals, and it can detect a unique muropeptide of iE-DAP (23). However, the NOD1 function in fish remains ambiguous because of the limited available experimental materials for studying the function of genes. NOD1 is structurally homologous with mammalian NOD1, which can detect a wide array of microbial components (48). Therefore, in this study, we have demonstrated that NOD1 can distinguish LPS through LRR domain and recruit RIPK2 to transfer the signal to induce inflammatory response in fish. This study was first to report that NOD1 can recognize LPS in teleost fish. These results elucidate the resistance of fish against bacterial infections. A theoretical basis is provided for future studies on the treatment of fish diseases.

## Author Contributions

Conceived and designed the experiments: TX and DB. Performed the experiments: DB, YW, TX, XL, YG, QC, and JC. Analyzed the data: TX and DB. Wrote the paper: TX, DB, and XL.

## Conflict of Interest Statement

The authors declare that the research was conducted in the absence of any commercial or financial relationships that could be construed as a potential conflict of interest.

## References

[1] Rietschel ET Kirikae T Schade FU Mamat U Schmidt G Loppnow H Bacterial endotoxin: molecular relationships of structure to activity and function. FASEB J (1994) 8:217–25. 10.1096/fasebj.8.2.8119492 8119492

[2] Schletter J Heine H Ulmer AJ Rietschel ET. Molecular mechanisms of endotoxin activity. Arch Microbiol (1995) 164:383–9. 10.1007/BF02529735 8588739

[3] Aderem A Ulevitch RJ. Toll-like receptors in the induction of the innate immune response. Nature (2000) 406:782–7. 10.1038/35021228 10963608

[4] Bohannon JK Hernandez A Enkhbaatar P Adams WL Sherwood ER. The immunobiology of toll-like receptor 4 agonists: from endotoxin tolerance to immunoadjuvants. Shock (2013) 40:451–62. 10.1097/SHK.0000000000000042 23989337 PMC3919163

[5] Frazão JB Errante PR Condino-Neto A Toll-like receptors’ pathway disturbances are associated with increased susceptibility to infections in humans. Arch Immunol Ther Exp (Warsz) (2013) 61:427–43. 10.1007/s00005-013-0243-0 24057516

[6] Liu Y Yin H Zhao M Lu Q. TLR2 and TLR4 in autoimmune diseases: a comprehensive review. Clin Rev Allergy Immunol (2014) 47:136–47. 10.1007/s12016-013-8402-y 24352680

[7] Newton K Dixit VM Signaling in innate immunity and inflammation. Cold Spring Harb Perspect Biol (2012) 4:829–41. 10.1101/cshperspect.a006049 PMC3282411 22296764

[8] Vanaja SK Russo AJ Behl B Banerjee I Yankova M Deshmukh SD Bacterial outer membrane vesicles mediate cytosolic localization of LPS and caspase-11 activation. Cell (2016) 165:1106–19. 10.1016/j.cell.2016.04.015 27156449 PMC4874922

[9] Shi JJ Zhao Y Wang YP Gao WQ Ding JJ Li P Inflammatory caspases are innate immune receptors for intracellular LPS. Nature (2014) 514:187–92. 10.1038/nature13683 25119034

[10] Hagar JA Powell DA Aachoui Y Ernst RK Miao EA. Cytoplasmic LPS activates caspase-11: implications in TLR4-independent endotoxic shock. Science (2013) 341:1250–3. 10.1126/science.1240988 24031018 PMC3931427

[11] Toranzo AE Magariños B Romalde JL A review of the main bacterial fish diseases in mariculture systems. Aquaculture (2005) 246:37–61. 10.1016/j.aquaculture.2005.01.002

[12] Swain P Nayak SK Nanda PK Dash S. Biological effects of bacterial lipopolysaccharide (endotoxin) in fish: a review. Fish Shellfish Immunol (2008) 25:191–201. 10.1016/j.fsi.2008.04.009 18603445

[13] Rebl A Goldammer T Seyfert HM. Toll-like receptor signaling in bony fish. Vet Immunol Immunopathol (2010) 134:139–50. 10.1016/j.vetimm.2009.09.021 19850357

[14] Su J Yang C Xiong F Wang Y Zhu Z. Toll-like receptor 4 signaling pathway can be triggered by grass carp reovirus and *Aeromonas hydrophila* infection in rare minnow *Gobiocypris rarus*. Fish Shellfish Immunol (2009) 27:33–9. 10.1016/j.fsi.2009.02.016 19264133

[15] Palti Y. Toll-like receptors in bony fish: from genomics to function. Dev Comp Immunol (2011) 35:1263–72. 10.1016/j.dci.2011.03.006 21414346

[16] Sepulcre MP Alcaraz-Perez F Lopez-Munoz A Roca FJ Meseguer J Cayuela ML Evolution of lipopolysaccharide (LPS) recognition and signaling: fish TLR4 does not recognize LPS and negatively regulates NF-kappaB activation. J Immunol (2009) 182:1836–45. 10.4049/jimmunol.0801755 19201835

[17] Wilmanski JM Petnicki-Ocwieja T Kobayashi KS. NLR proteins: integral members of innate immunity and mediators of inflammatory diseases. J Leukoc Biol (2008) 83:13–30. 10.1189/jlb.0607402 17875812 PMC3256237

[18] Geddes BJ Wang L Huang WJ Lavellee M Manji GA Brown M Human CARD4 protein is a novel CED-4/Apaf-1 cell death family member that activates NF-κB. J Biol Chem (1999) 274:12955–8. 10.1074/jbc.274.19.12955 10224040

[19] Stein C Caccamo M Laird G Leptin M. Conservation and divergence of gene families encoding components of innate immune response systems in zebrafish. Genome Biol (2007) 8:R251. 10.1186/gb-2007-8-11-r251 18039395 PMC2258186

[20] Xie J Hodgkinson JW Katzenback BA Kovacevic N Belosevic M. Characterization of three Nod-like receptors and their role in antimicrobial responses of goldfish (*Carassius auratus* L.) macrophages to *Aeromonas salmonicida* and *Mycobacterium marinum*. Dev Comp Immunol (2013) 39:180–7. 10.1016/j.dci.2012.11.005 23194927

[21] Hou QH Yi SB Ding X Zhang HX Sun Y Zhang Y Differential expression analysis of nuclear oligomerization domain proteins NOD1 and NOD2 in orange-spotted grouper (*Epinephelus coioides*). Fish Shellfish Immunol (2012) 33:1102–11. 10.1016/j.fsi.2012.08.015 22982325

[22] Li J Gao Y Xu T. Comparative genomic and evolution of vertebrate NOD1 and NOD2 genes and their immune response in miiuy croaker. Fish Shellfish Immunol (2015) 46:387–97. 10.1016/j.fsi.2015.06.026 26108036

[23] Girardin SE Boneca IG Carneiro LA Antignac A Jehanno M Viala J Nod1 detects a unique muropeptide from Gram-negative bacterial peptidoglycan. Science (2003) 300:1584–7. 10.1126/science.1084677 12791997

[24] Inohara N Ogura Y Chen FF Muto A Nuñez G. Human Nod1 confers responsiveness to bacterial lipopolysaccharides. J Biol Chem (2001) 276:2551–4. 10.1074/jbc.M009728200 11058605

[25] Inohara N Koseki T Lin J del Peso L Lucas PC Chen FF An induced proximity model for NF-κB activation in the Nod1/RICK and RIP signaling pathways. J Biol Chem (2000) 275:27823–31. 10.1074/jbc.M003415200 10880512

[26] Bi D Gao Y Chu Q Cui J Xu T. NOD1 is the innate immune receptor for iE-DAP and can activate NF-κB pathway in teleost fish. Dev Comp Immunol (2017) 76:238–46. 10.1016/j.dci.2017.06.012 28655577

[27] MacKenzie SA Roher N Boltaña S Goetz FW. Peptidoglycan, not endotoxin, is the key mediator of cytokine gene expression induced in rainbow trout macrophages by crude LPS. Mol Immunol (2010) 47:1450–7. 10.1016/j.molimm.2010.02.009 20304498

[28] Berczi I Bertok L Bereznai T Comparative studies on the toxicity of *Escherichia coli* lipopolysaccharide endotoxin in various animal species. Can J Microbiol (1966) 12:1070–1. 10.1139/m66-143 5339644

[29] Chu Q Sun Y Cui J Xu T. MicroRNA-3570 modulates the NF-κB pathway in teleost fish by targeting MyD88. J Immunol (2017) 198:3274–82. 10.4049/jimmunol.1602064 28250156

[30] Chu Q Sun Y Cui J Xu T Inducible microRNA-214 contributes to the suppression of NF-κB–mediated inflammatory response via targeting MyD88 gene in fish. J Biol Chem (2017) 292:5282–90. 10.1074/jbc.M117.777078 28235799 PMC5392675

[31] Xu T Xu G Che R Wang R Wang Y Li J The genome of the miiuy croaker reveals well-developed innate immune and sensory systems. Sci Rep (2016) 6:21902. 10.1038/srep21902 26902509 PMC4763219

[32] Zhao X Chu Q Cui J Huo R Xu T. IRF9 as a negative regulator involved in TRIF-mediated NF-κB pathway in a teleost fish, *Miichthys miiuy*. Mol Immunol (2017) 85:123–9. 10.1016/j.molimm.2017.02.009 28236773

[33] Huo R Zhao X Han J Xu T. Genomic organization, evolution and functional characterization of soluble toll-like receptor 5 (TLR5S) in miiuy croaker (*Miichthys miiuy*). Fish Shellfish Immunol (2018) 80:109–44. 10.1016/j.fsi.2018.05.048 29857132

[34] Zhou J Zhao S Fang WH Zhou JF Zhang JX Ma H Newly identified invertebrate-type lysozyme (Splys-i) in mud crab (*Scylla paramamosain*) exhibiting muramidase-deficient antimicrobial activity. Dev Comp Immunol (2017) 74:154–66. 10.1016/j.dci.2017.04.017 28438599

[35] Hou ZG Wang Y Hui K Fang WH Zhao S Zhang JX A novel anti-lipopolysaccharide factor SpALF6 in mud crab *Scylla paramamosain* exhibiting different antimicrobial activity from its single amino acid mutant. Dev Comp Immunol (2017) 72:44–56. 10.1016/j.dci.2017.02.009 28232132

[36] Rieu I Powers SJ Real-time quantitative RT-PCR: design, calculations, and statistics. Plant Cell (2009) 21:1031–3. 10.1105/tpc.109.066001 19395682 PMC2685626

[37] Hopkins PA Sriskandan S. Mammalian toll-like receptors: to immunity and beyond. Clin Exp Immunol (2005) 140:395–407. 10.1111/j.1365-2249.2005.02801.x 15932500 PMC1809390

[38] Wang Y Bi X Chu Q Xu T Discovery of toll-like receptor 13 exists in the teleost fish: Miiuy croaker (Perciformes, Sciaenidae). Dev Comp Immunol (2016) 61:25–33. 10.1016/j.dci.2016.03.005 26952767

[39] Boudinot P Zou J Ota T Buonocore F Scapigliati G Canapa A A tetrapod-like repertoire of innate immune receptors and effectors for *coelacanths*. J Exp Zoolog B Mol Dev Evol (2014) 322:415–37. 10.1002/jez.b.22559 24482296

[40] Wang Y Li J Han J Shu C Xu T. Identification and characteristic analysis of TLR28: a novel member of the TLR1 family in teleost. Dev Comp Immunol (2016) 62:102–7. 10.1016/j.dci.2016.05.001 27155354

[41] Hwang SD Kondo H Hirono I Aoki T. Molecular cloning and characterization of toll-like receptor 14 in Japanese flounder, *Paralichthys olivaceus*. Fish Shellfish Immunol (2011) 30:425–9. 10.1016/j.fsi.2010.08.005 20728538

[42] Philpott DJ Yamaoka S Israël A Sansonetti PJ Invasive *Shigella flexneri* activates NF-κB through a lipopolysaccharide-dependent innate intracellular response and leads to IL-8 expression in epithelial cells. J Immunol (2000) 165:903–14. 10.4049/jimmunol.165.2.903 10878365

[43] Funda DP Tučková L Farré MA Iwase T Moro I Tlaskalová-Hogenová H. CD14 is expressed and released as soluble CD14 by human intestinal epithelial cells in vitro: lipopolysaccharide activation of epithelial cells revisited. Infect Immun (2001) 69:3772–81. 10.1128/IAI.69.6.3772-3781.2001 11349042 PMC98389

[44] Fukata M Michelsen KS Eri R Thomas LS Hu B Lukasek K Toll-like receptor-4 is required for intestinal response to epithelial injury and limiting bacterial translocation in a murine model of acute colitis. Am J Physiol Gastrointest Liver Physiol (2005) 288:G1055–65. 10.1152/ajpgi.00328.2004 15826931

[45] Hughes AL. Evolutionary relationships of vertebrate NACHT domain-containing proteins. Immunogenetics (2006) 58:785–91. 10.1007/s00251-006-0148-8 17006665

[46] Hibino T Loza-Coll M Messier C Majeske AJ Cohen AH Terwilliger DP The immune gene repertoire encoded in the purple sea urchin genome. Dev Biol (2006) 300:349–65. 10.1016/j.ydbio.2006.08.065 17027739

[47] Laing KJ Purcell MK Winton JR Hansen JD. A genomic view of the NOD-like receptor family in teleost fish: identification of a novel NLR subfamily in zebrafish. BMC Evol Biol (2008) 8:42. 10.1186/1471-2148-8-42 18254971 PMC2268669

[48] Dixon MS Golstein C Thomas CM Van der Biezen EA Jones JD. Genetic complexity of pathogen perception by plants: the example of Rcr3, a tomato gene required specifically by Cf-2. Proc Natl Acad Sci U S A (2000) 97:8807–14. 10.1073/pnas.97.16.8807 10922039 PMC34016

