# Supplementary material for: Recognition of Lipopolysaccharide and Activation of NF-κB by Cytosolic Sensor NOD1 in Teleost Fish
Source: Front Immunol. 2018 Jun 26;9:1413. doi: 10.3389/fimmu.2018.01413 (PMC6036275; doi:10.3389/fimmu.2018.01413)
Supplement: Supplementary file 1 [file Image_1.PDF]

Supplementary material

Recognition of lipopolysaccharide and activation of NF-κB by cytosolic sensor NOD1 in teleost fish

Dekun Bi[1,2,3], Yue Wang[4], Yunhang Gao[5], Xincang Li[4], Qing Chu[1,2,6], Junxia Cui[1,2,3], Tianjun Xu[1,2,3,6,*]

1. Key Laboratory of Exploration and Utilization of Aquatic Genetic Resources (Shanghai Ocean University), Ministry of Education, 201306, China

2. National Pathogen Collection Center for Aquatic Animals, Shanghai Ocean University, 201306, China

3. Laboratory of Fish Biogenetics & Immune Evolution, College of Marine Science, Zhejiang Ocean University, Zhoushan, 316022, China

4.East China Sea Fisheries Research Institute, Chinese Academy of Fishery Sciences, shanghai, 2 00090, China

5. College of Animal Science and Veterinary Medicine, Jilin Agriculture University, Changchun, 130118, China

6. International Research Center for Marine Biosciences at Shanghai Ocean University, Ministry of Science and Technology, 201306, China

*Corresponding author. Dr. Tianjun Xu

E-mail: tianjunxu@163. com

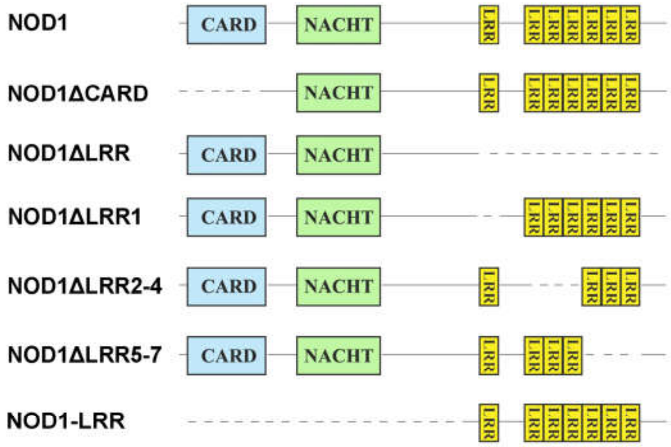

Supplementary Figure 1. The schematic of NOD1 mutant plasmids**.**

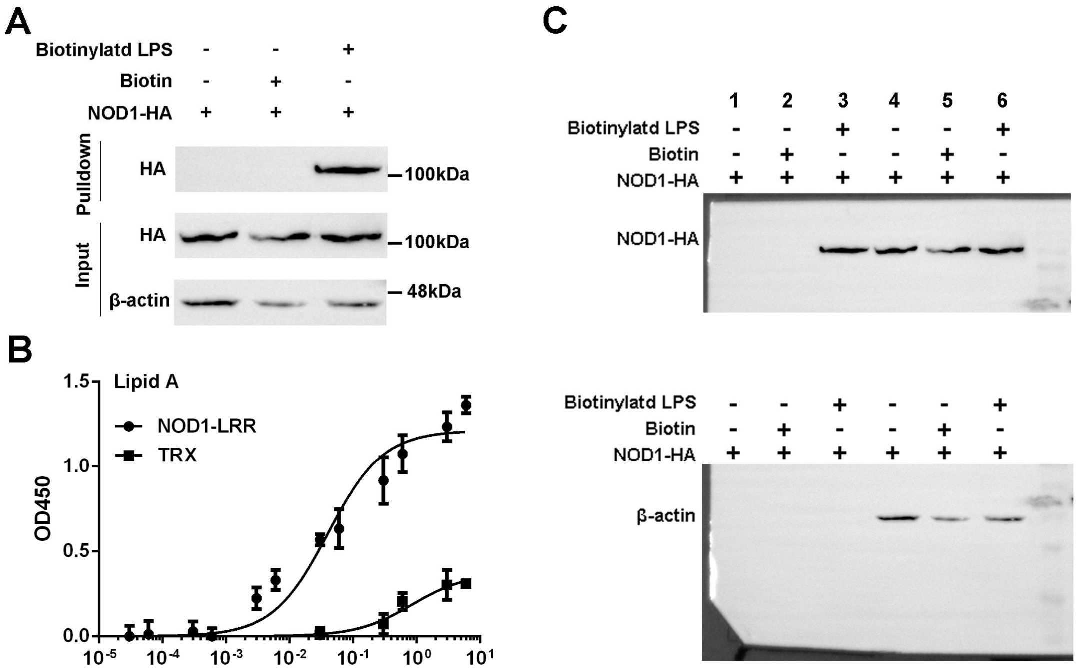

Supplementary Figure 2. Supplementary experiment of pulldown assay and the original images. (**A**) HEK293 cells were transfected of NOD1 expression plasmids, after transfection of 36h, stimulate cells with Biotinylatd LPS or Biotin, after stimulated with 12h, collect cells and perform pulldown assay. (**B**) Binding activity analysis of miiuy croaker NOD1-LRR and TRX to synthetic Lipid A (tlrl-mpls, InvivoGen). (**C**)The original images of the **Supplementary Figure 2A** experiment. The lines of 1, 2 and 3 were the protein sample that after perform pulldown assay. And the lines of 4, 5 and 6 were the protein sample before perform pulldown assay.

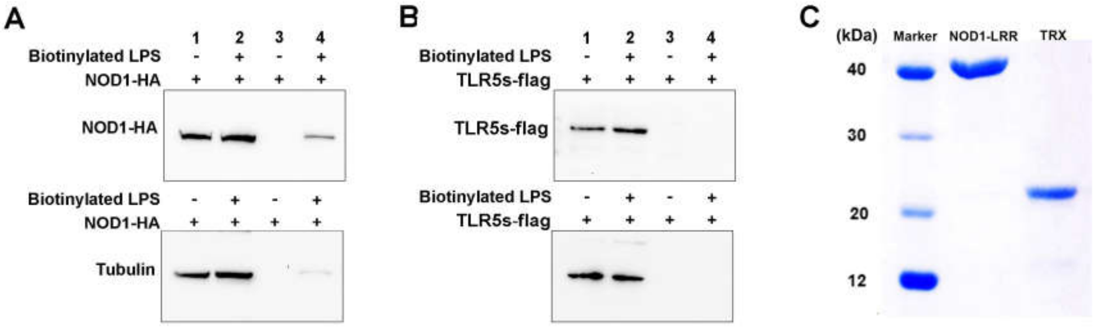

Supplementary Figure 3. The original images of the experiment of Fig. 5A and the expression analysis of NOD1-LRR and TRX proteins. (**A**) and (**B**) The original images of the pulldown assay of Fig. 5A. The lines of 1 and 2 were the protein sample before perform pulldown assay. The lines of 3 and 4 were the protein sample that after perform pulldown assay. (**C**) SDS-PAGE analysis of recombinant Miiuy croaker NOD1-LRR and TRX which expressed in E. coli cells, lane NOD1-LRR and TRX was represent total protein of E. coli after induction with IPTG and purified by Ni-NTA His Bind Resin. The purified NOD1-LRR comprised an LRR domain (theoretical molecular weight (MW) of 24.1 kDa) and an approximately 17.8 kDa TRX Tag protein expressed by the plasmid pET-32a, so the size of the recombinant protein was in agreement with the position (approximately 41.9 kDa). TRX was purified as the control protein and had two His-tagged regions and other necessary amino acids, except the TRX Tag region. The theoretical MW of this control protein is 21.0 kDa.

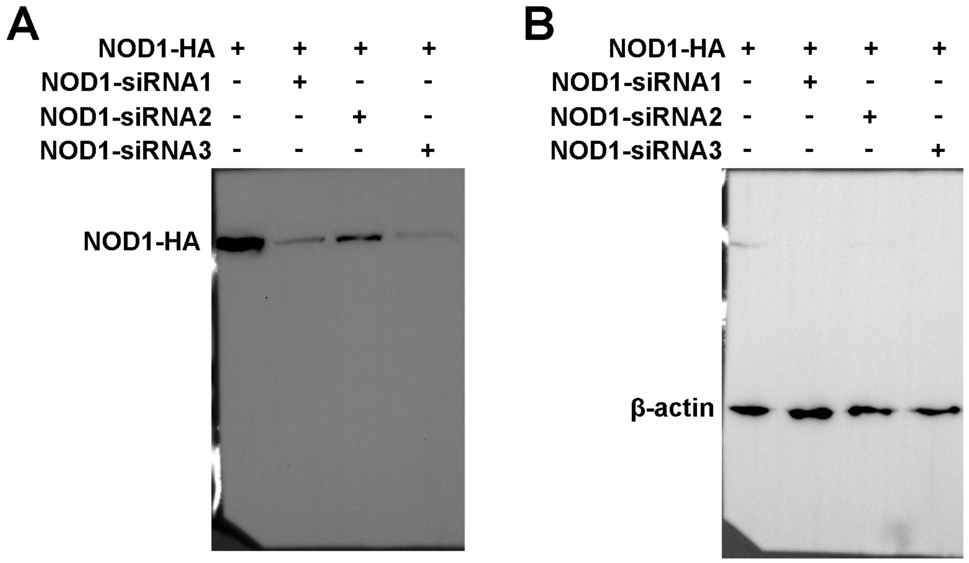

Supplementary Figure 4. The original images of the experiment of Fig. 6A. (**A**) PVDF membrane was incubated by using anti-HA monoclonal antibody. (**B**) After removing the antibodies on the membrane by using stripping buffer (Beyotime, P0025B), PVDF membrane was incubated by using anti-β-actin monoclonal antibody.

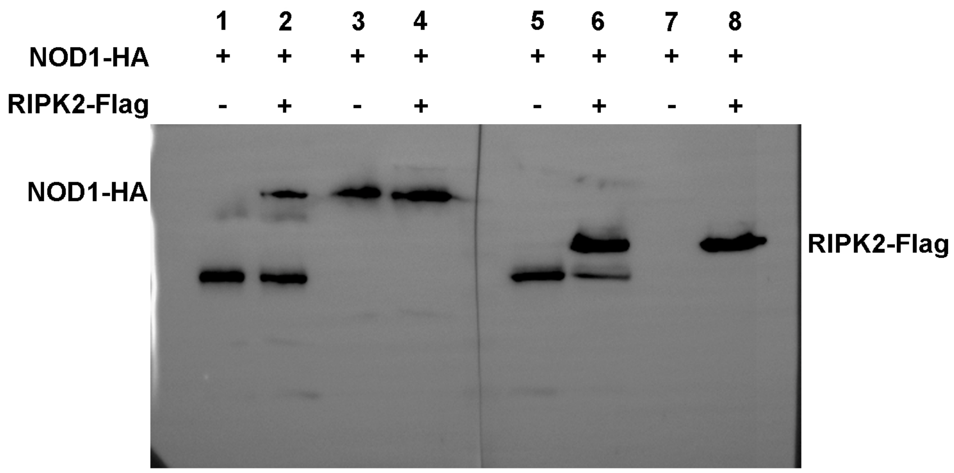

Supplementary Figure 5. The original images of the experiment of Fig. 8C. The left half of the PVDF membrane was incubated by using anti-HA monoclonal antibody. The right half of the PVDF membrane was incubated by using anti-Flag monoclonal antibody. The lines of 1, 2, 5 and 6 were the protein sample after perform Immunoprecipitation experiment, and the lines of 3, 4, 7 and 8 were the protein sample before perform Immunoprecipitation experiment.

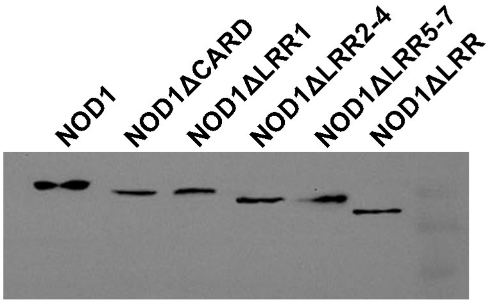

Supplementary Figure 6. The original images of the experiment of Fig. 9A. HEK293 cells were transfected of NOD1 wild type plasmids and different mutant plasmids, after transfection of 48h, collection of cells and perform Western blotting assay.

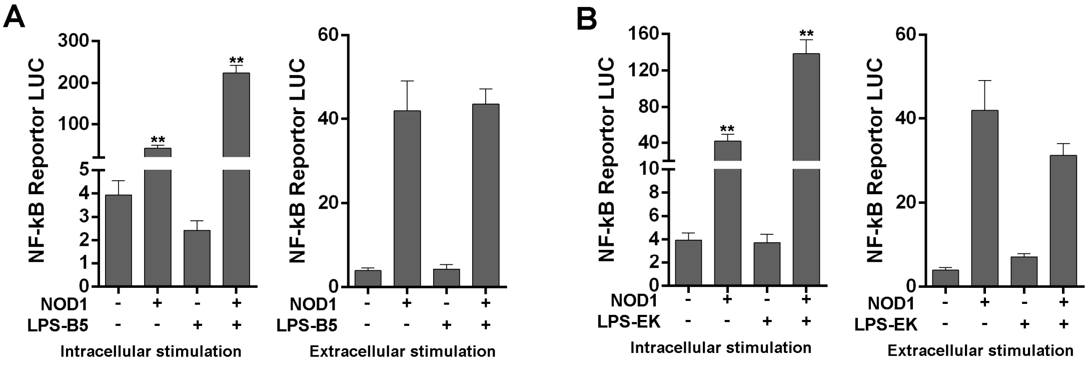

Supplementary Figure 7. The supplementary experiment of stimulation. HEK293 cells were co-transfecting with NOD1 expression plasmids and NF-κB reporter plasmids, pRL-TK plasmids as the internal control. Then transfection of LPS-B5 **(A)** and LPS-EK **(B)** into cells to perform intracytoplasmic stimulation experiments or directly added LPS-B5 and LPS-EK into cell culture medium for extracellular stimulation experiments.
